# Indirect protection of children from SARS-CoV-2 infection through parental vaccination

**DOI:** 10.1126/science.abm3087

**Published:** 2022-01-27

**Authors:** Samah Hayek, Galit Shaham, Yatir Ben-Shlomo, Eldad Kepten, Noa Dagan, Daniel Nevo, Marc Lipsitch, Ben Y. Reis, Ran D. Balicer, Noam Barda

**Affiliations:** ^1^ Clalit Research Institute, Clalit Health Services, Ramat Gan, Israel.; ^2^ Software and Information Systems Engineering, Ben Gurion University, Be’er Sheva, Israel.; ^3^ Department of Biomedical Informatics, Harvard Medical School, Boston, MA, USA.; ^4^ The Ivan and Francesca Berkowitz Family Living Laboratory Collaboration at Harvard Medical School and Clalit Research Institute, Boston, MA, USA.; ^5^ Department of Statistics and Operations Research, Tel Aviv University, Tel Aviv, Israel.; ^6^ Center for Communicable Disease Dynamics, Department of Epidemiology, and Department of Immunology and Infectious Diseases, Harvard T.H. Chan School of Public Health, Boston, MA, USA.; ^7^ Predictive Medicine Group, Computational Health Informatics Program, Boston Children’s Hospital, Boston, MA, USA.; ^8^ School of Public Health, Faculty of Health Sciences, Ben-Gurion University of the Negev, Beer-Sheba, Israel.

## Abstract

Children not vaccinated against severe acute respiratory syndrome coronavirus 2 (SARS-CoV-2) may still benefit from vaccines through protection from vaccinated contacts. We estimated the protection provided to children through parental vaccination with the BNT162b2 vaccine. We studied households without prior infection consisting of two parents and unvaccinated children, estimating the effect of parental vaccination on the risk of infection for unvaccinated children. We studied two periods separately—an early period (17 January 2021 to 28 March 2021; Alpha variant, two doses versus no vaccination) and a late period (11 July 2021 to 30 September 2021; Delta variant, booster dose versus two vaccine doses). We found that having a single vaccinated parent was associated with a 26.0 and a 20.8% decreased risk in the early and late periods, respectively, and having two vaccinated parents was associated with a 71.7 and a 58.1% decreased risk, respectively. Thus, parental vaccination confers substantial protection on unvaccinated children in the household.

## Protection, whether direct or not

Vaccination provides both direct protection of vaccinated individuals and indirect protection of individuals living in vaccinated communities. Two studies based on data from Israel investigated the efficacy and indirect protection of the Pfizer/BioNTech messenger RNA vaccine (see the Perspective by Dean and Halloran). Prunas *et al*. used statistical approaches to analyze transmission in households from June 2020 to July 2021. People who were vaccinated and subsequently infected were less infectious than unvaccinated persons. Moreover, less transmission occurred within households with vaccinated members than in those with unvaccinated individuals. However, the ability of the vaccine to prevent transmission waned with time and with the advent of the Delta variant. Hayek *et al*. investigated whether older and vaccinated household members reduced the risk of infection to younger children who are as yet ineligible for vaccination. Regardless of household size, parental vaccination substantially reduced the risk of children up to 12 years old becoming infected. This indirect effect will protect children from risk of severe disease and reduce the propagation of transmission chains. —CA

## Teaser

Parental Covid-19 vaccination confers substantial protection for unvaccinated children in the household.

Since December 2019, severe acute respiratory syndrome coronavirus 2 (SARS-CoV-2) has spread globally (*1*), resulting in more than 200 million confirmed infections and more than 4 million deaths (*2*). COVID-19 vaccines serve a critical role in combating the spread of the pandemic. Vaccination exerts its effects through both direct protection of vaccinated individuals and indirect protection of individuals living in vaccinated environments (*3*).

Households have specific importance in the context of infectious disease dynamics. Several epidemiological studies have reported that a substantial amount of COVID-19 transmission occurs in settings that include close and prolonged contact, such as households (*4*–*6*)_._ The importance of households in SARS-CoV-2 transmission was highlighted in a recent meta-analysis, in which the secondary attack rate was found to be 19.0% [95% confidence interval (CI): 16.2%, 22.0%] (*7*). The central role of households in SARS-CoV-2 transmission allows them to be used as alternatives to larger clusters for estimating the direct and indirect effects of vaccines (*3*)_._

Unlike the direct effect of the BNT162b2 mRNA COVID-19 vaccine, which has been extensively explored in clinical trials (*8*) and observational studies (*9*, *10*), the indirect effect of the vaccine has not received as much attention. Previous studies have shown that a single vaccinated household member confers modest protection [42.9% (95% CI: 22.3%, 58.1%), 10 weeks after the first dose] against SARS-CoV-2 infection on other unvaccinated adult household members (*11*). A study from Israel has shown that vaccination reduces the risk of infection and of transmission once an infection is introduced into the household and that unvaccinated spouses of health care workers are protected by their spouse’s vaccination (*12*). A different study evaluated the indirect effect at a different level, using 177 geographical communities in Israel, and showed that higher rates of vaccination in each community were associated with a substantial decline in infections among a cohort of unvaccinated individuals aged 16 years or younger (*13*). In general, previous studies concerning indirect effects of vaccination had small sample sizes, included only specific populations (e.g., health care workers), did not adjust for certain important confounders, only covered a single period and disease variant, and did not explore the mechanism of the indirect effect.

In Israel, the BNT162b2 mRNA COVID-19 vaccine was authorized in December 2020 for individuals aged 16 years and older. In May 2021, this authorization was extended to children and adolescents aged 12 years and older and, in November 2021, to children aged 5 years and older. Third-dose booster shots were initiated in Israel on 11 July 2021 and were gradually extended to cover the entire population—who had received the second dose at least 5 months prior—over the month of August. In parallel, from December 2020 to March 2021, Israel underwent a third wave of the COVID-19 pandemic, in which the Alpha variant was dominant. This wave was accompanied by a nationwide lockdown that included closure of schools and limitation of social activities. A fourth wave occurred in Israel from June to October 2021, this time dominated by the Delta variant. No lockdowns were in effect during this wave; however, during the months of July and August, the schools were closed for summer vacation. Throughout 2021, COVID-19 polymerase chain reaction (PCR) tests were freely available nationwide and targeted sampling was performed in schools in which a teacher or a child were found to be infected. In Israel (*14*), as in Europe (*15*) and the US (*16*), the younger age groups remain the least vaccinated.

In this study, we use the integrated data repositories of Israel’s largest health care organization to estimate the indirect vaccine effectiveness (VE) of the BNT162b2 mRNA COVID-19 vaccine on unvaccinated children within households. We perform this analysis over two time periods: an early period (17 January 2021 through 28 March 2021) in children <16 years old when the Alpha variant was dominant, in which we compare households with parents who were vaccinated with the primary vaccine series with households with unvaccinated parents, and a late period (11 July 2021 through 30 September 2021) in children <11 years old when the Delta variant was dominant, in which we compare households with parents who were vaccinated with a booster dose with households in which parents were previously vaccinated with two vaccine doses but have not received the booster dose. In each period, we assess the change in the risk of SARS-CoV-2 infection among susceptible children in the household (who are not eligible for vaccination) associated with the vaccination of one or both parents. Furthermore, in each period, we explore two of the mechanisms mediating this effect by estimating the decrease in risk that a vaccinated parent would be infected (direct VE) and the decrease in risk that a vaccinated infected parent would then proceed to infect a susceptible child [household infectiousness, or secondary attack rate (SAR)].

The early period of the study included 400,733 unvaccinated subjects (children and adolescents) from 155,305 distinct households who contributed 2,116,306 person-weeks (defined as one week of follow-up for one subject) of follow-up (fig. S1A). The median age of the children was 6 years old [interquartile range (IQR): 3, 9], and 52% of subjects were male. The late period of the study included 181,307 unvaccinated children from 76,621 distinct households who contributed 1,089,191 person-weeks of follow-up (fig. S1B). The median age of the children was 5 years old (IQR: 2, 7), and 52% of subjects were male.

Baseline demographic and clinical characteristics of the subjects in each time period are shown in Table 1. A more detailed description, including all potential confounders stratified by parental vaccination status, is presented in table S1. A time series of the cases observed in our study (the epidemic curve) during both periods, stratified by age group, are presented in fig. S2. Table S2 describes the differences between the infected and uninfected subjects in both study periods.

**Table 1.** Descriptive statistics of the study population. The study population includes susceptible children under the age of vaccination eligibility and residing in the households included in the study. The early period was 17 January 2021 to 28 March 2021. The late period was 11 July 2021 to 30 September 2021. NA, not applicable.

**Characteristic**	**Early period (*N* = 400,733)**	**Late period (*N* = 181,307)**
Median age (IQR)	6 (3, 9)	5 (2, 7)
Median household size (IQR)	5 (4, 6)	5 (4, 5)
*Age (*N*, %)*		
0-2	81,672 (20%)	47,710 (26%)
3-6	135,230 (34%)	75,278 (42%)
7-12*	149,917 (37%)	58,319 (32%)
13-15	33,914 (8.5%)	NA
*Sex (*N*, %)*		
Female	194,272 (48%)	87,913 (48%)
Male	206,461 (52%)	93,394 (52%)
*Population group (*N*, %)*		
Arabs	101,557 (25%)	32,484 (18%)
General	277,444 (69%)	140,222 (77%)
Ultra-Orthodox Jewish	21,732 (5.4%)	8,601 (4.7%)
*Socioeconomic status (*N*, %)*		
Low	223,108 (56%)	88,023 (49%)
Medium	162,833 (41%)	87,380 (48%)
High	14,792 (3.7%)	5,904 (3.3%)
*Household size (*N*, %)*		
3	20,127 (5.0%)	11,936 (6.6%)
4	107,549 (27%)	63,866 (35%)
5	157,379 (39%)	73,150 (40%)
6	80,423 (20%)	24,878 (14%)
7	35,255 (8.8%)	7,477 (4.1%)
*Residence type (*N*, %)*		
Large city	127,887 (32%)	62,991 (35%)
Small city	149,260 (37%)	67,702 (37%)
Town	76,764 (19%)	28,817 (16%)
Rural	31,555 (7.9%)	13,888 (7.7%)
Kibbutz (communal residence)	15,267 (3.8%)	7,909 (4.4%)
*Chronic conditions (*N*, %)*		
Obesity	24,780 (6.2%)	9,524 (5.3%)
Cardiovascular conditions	1,833 (0.5%)	460 (0.3%)
Pulmonary disease	47,823 (12%)	18,779 (10%)
Type 2 diabetes	1,833 (0.5%)	636 (0.4%)
Hypertension	619 (0.2%)	238 (0.1%)
Active malignancy	240 (<0.1%)	135 (<0.1%)

*The late period includes children up to age 11.

**Fig. 1.**
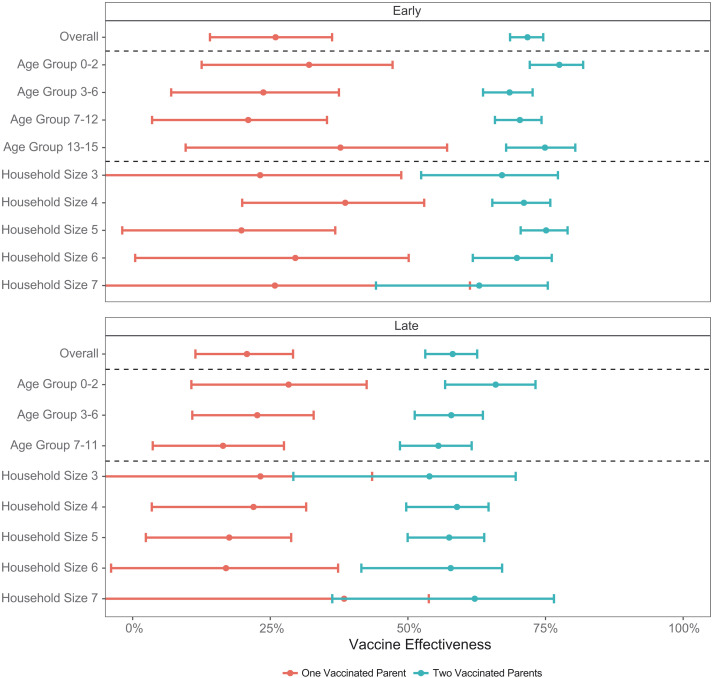
Indirect effect of the BNT162b2 mRNA COVID-19 vaccine by age group and household size. Indirect VE (one minus the incidence rate ratio) of one vaccinated parent and two vaccinated parents on the probability of infection of a susceptible child within the household, overall and within age group and household size categories. Points represent the point estimates, and error bars represent the 95% CIs. The top part shows the early study period (vaccination with two doses at least 7 days prior versus no vaccination; Alpha variant), and the bottom part shows the late study period (receipt of the booster dose versus no receipt of the booster dose; Delta variant). The numeric results included in this figure are presented in table S3.

During the early period, focusing on the Alpha variant and comparing parents vaccinated with the primary vaccine series with unvaccinated parents, a single vaccinated parent was associated with a 26.0% (95% CI: 14.0%, 36.2%) decreased risk of infection for children living in the same household, and two vaccinated parents were associated with a 71.7% (68.6%, 74.6%) decreased risk of infection. This effect was fairly uniform across subject age groups and household sizes. For example, the adjusted VE was 67.1% (52.4%, 77.3%) for a household of size 3 in which both parents were vaccinated and 62.9% (44.2%, 75.4%) for a household of size 7 in which both parents were vaccinated (Fig. 1 and table S3).

During the late period, focusing on the Delta variant and comparing parents vaccinated with a third (booster) dose with parents who received only two doses at least 5 months prior, a single boosted parent was associated with a 20.8% (95% CI: 11.4%, 29.1%) decreased risk for infection, whereas two boosted parents were associated with a 58.1% (53.1%, 62.6%) decreased risk for infection. Some heterogeneity of the effect was observed between age groups and household sizes. For example, the adjusted VE was 65.9% (56.7%, 73.2%) for a subject aged 0 to 2 years living with two boosted parents, and the adjusted VE was 55.5% (48.6%, 61.6%) for a subject aged 7 to 11 years living with two boosted parents (Fig. 1 and table S3). In both periods, plots of the predicted versus observed incidence rates indicate a good model fit (fig. S3).

Analysis of the direct effect of the BNT162b2 mRNA COVID-19 vaccine on the risk of parental infection estimated a reduction of 94.4% (95% CI: 93.2%, 95.4%) in the risk of documented infection during the early period (Alpha variant) and 86.3% (83.4%, 88.6%) in the risk of documented infection during the late period (Delta variant) among fully vaccinated adults (Table 2). Full vaccination of an infected parent was associated with a 72.1% (36.6%, 89.3%) decreased odds of infection of one or more susceptible children in the household from that parent during the early period and a 79.6% (55.9%, 91.8%) decreased odds of transmission from a boosted, infected parent to one or more susceptible children during the late period (Table 3), in both cases adjusting for the vaccination status of the other parent.

**Table 2.** Direct effect of BNT162b2 mRNA COVID-19 vaccine. Direct VE is the reduction in the probability of infection of a fully vaccinated parent compared with an unvaccinated or unboosted parent, defined as one minus the incidence rate ratio. During the early period, full vaccination was defined as the receipt of two doses at least 7 days prior (compared with no vaccination), and the dominant variant was Alpha. During the late period, full vaccination was defined as receipt of a third dose at least 7 days prior (compared with receipt of only two doses at least 5 months prior), and the dominant variant was Delta. Analysis was performed as per the main analysis, this time using parental infection as the outcome. The model was adjusted for individual- and household-level characteristics. See table S1 for the full list.

** Characteristic**	**Early period**	**Late period**
Direct VE (95% CI)	94.4% (93.2%, 95.4%)	86.3% (83.4%, 88.6%)

**Table 3.** Secondary transmission risk. The secondary attack rate (SAR) from an infected parent to susceptible children in the household by parent vaccination status. The unit of observation for this analysis consisted of households in which a parent (the index parent) was infected with SARS-CoV-2. The exposure was the vaccination status of the index parent. The outcome was infection of at least one child in the household at days 3 to 8 after diagnosis of the index parent. To maintain a well-defined point of entry of the infection, we excluded households in which the parent who is not the index parent or a child was diagnosed on days 0 to 2 after diagnosis of the index parent. During the early period, full vaccination was defined as the receipt of two doses at least 7 days prior (compared with no vaccination), and the dominant variant was Alpha. During the late period, full vaccination was defined as receipt of a third dose at least 7 days prior (compared with receipt of only two doses at least 5 months prior), and the dominant variant was Delta. The adjusted estimate was derived from a logistic regression model adjusted for all the household-level characteristics and the vaccination status of the nonindex parent.

** Characteristic**	**Early period**	**Late period**
SAR from a vaccinated or boosted parent (%)	9.0%	9.3%
SAR from an unvaccinated or unboosted parent (%)	24.7%	31.1%
1 − adjusted odds ratio (95% CI)	72.1% (36.6%, 89.3%)	>79.6% (55.9%, 91.8%)

**Fig. 2.**
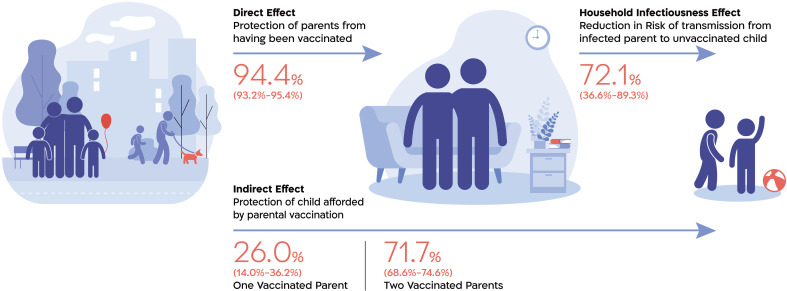
Mechanism of disease transmission. An illustration showing the indirect effect of parental vaccination on children’s risk of SARS-CoV-2 infection and two of its composite parts: the direct effect of vaccination on the parents (estimated as the incidence rate ratio of parental infection between vaccinated and unvaccinated parents) and the risk of transmission from an infected parent to his or her children (estimated as the odds ratio of an infected parent infecting at least one child in the household). We do not expect the indirect risk to equal the product of the direct risk and infectiousness because children may also be infected outside of the household or, potentially, through the other parent. Estimates shown are from the early study period, in which parents vaccinated with two vaccine doses at least 7 days prior were compared with unvaccinated parents and the dominant variant was Alpha.

Figure 2 shows a schematic representation of the mechanism by which the direct protection of the parent and the reduction in the secondary attack rate make up the indirect protection observed for the children. In the sensitivity analysis using bacterial diarrhea as a negative control outcome, the association (VE) was −14% (95% CI: −49%, 13%) for one vaccinated parent and −16% (−37%, 1.8%) for two vaccinated parents (table S4).

In this study, we estimated the indirect protective effect of vaccinating parents with the BNT162b2 mRNA COVID-19 vaccine on their children’s risk of SARS-CoV-2 infection in households without prior infection. This estimation was performed for both the primary vaccine series during a period in which the Alpha variant was dominant and the vaccine booster dose during a period in which the Delta variant was dominant. In both periods, we found that parental vaccination substantially reduced the risk of children being infected with SARS-CoV-2, though the effect was somewhat smaller during the late period. Although this smaller effect could result from heterogeneity, as the populations are different in composition, it more likely stems from unboosted parents still being somewhat protected from the first two vaccine doses, which makes the relative effect of the additional booster vaccination dose smaller. Notably, we found the effect for two vaccinated parents to be substantially larger than that for a single vaccinated parent in both periods (26.0% versus 71.7% in the early period; 20.8% versus 58.1% in the late period). This emphasizes that even a single unvaccinated parent remains an important vector for introducing infections into the household.

Previous findings have also shown a substantial indirect effect of SARS-CoV-2 vaccines. A study focusing on unvaccinated spouses of health care workers found the indirect effect to be 43% (95% CI: 23%, 58%) 10 weeks after receipt of the first vaccine dose (*11*). A study from Israel found that once COVID-19 is introduced into a household, vaccination reduces infectivity by 78% (30%, 94%) (*12*). Another study from Israel using geographical areas to estimate the community-level protection resulting from vaccinated individuals found that, on average, for every absolute 20% increase in the number of vaccinated individuals, the positive test fraction of the unvaccinated population decreased by a factor of ~2 (*13*). In general, it is difficult to directly compare the findings of these studies with the current study because of the different designs, adjustments, and exposure definitions.

The present study focused on the indirect benefits of vaccinated parents for unvaccinated children. Indirect vaccine effects are mediated by two main mechanisms: (i) by protecting potential contacts, vaccination reduces the likelihood that subjects will encounter an infectious individual and (ii) vaccination may reduce the infectiousness of vaccinated individuals who do acquire the infection (*17*, *18*). We explored these two mechanisms by estimating the direct effect of parental vaccination on parental infection as well as the vaccination-related change in the risk of infection from an infected parent to a susceptible child. We found the direct effect of parental vaccination with two vaccine doses to be 94.4% (95% CI: 93.2%, 95.4%) for acquiring a documented infection with the Alpha variant and the direct effect of a booster dose to be 86.3% (83.4%, 88.6%) for acquiring a documented infection with the Delta variant. This high effectiveness when comparing parents who have received the booster dose with those who have not also hints at waning immunity after the second dose. Furthermore, we found that infectiousness to the children in the household from an infected parent vaccinated with two doses is reduced by 72.1% (36.6%, 89.3%) compared with the infectiousness of an unvaccinated parent, and infectiousness from a booster-vaccinated parent is reduced by 79.6% (55.9%, 91.8%) compared with that of a parent who did not receive the booster vaccination dose, in each case adjusting for the vaccination status of the other parent. It should be emphasized that we should not expect the indirect risk to be equal to the product of the direct risk and the infectiousness because children may also be infected outside of the household or, potentially, through the other parent. The estimated direct VE of the parents is consistent with previous literature (*9*, *19*) as are the results concerning the reduced SAR (*12*).

To detect possible bias originating from uncontrolled confounding, we performed an analysis using a negative control outcome (NCO) (*20*, *21*), bacterial diarrhea. Bacterial diarrhea was chosen because it plausibly shares confounders (e.g., health-related behavior and hygiene) with the outcome of interest but should not be affected by the exposure of interest (SARS-CoV-2 vaccine). This analysis did not detect substantial effects, further strengthening our findings and reducing the possibility of meaningful unmeasured confounding.

The protective effect of parental vaccination on children’s risk described in this study has particular importance for several reasons: First, although children often experience asymptomatic or mild disease when infected with SARS-CoV-2, some do experience severe disease (*22*, *23*) and enduring postinfection symptoms (known as Long Covid) (*24*), particularly when suffering from some degree of immunosuppression (*25*). Second, because of the important role of households in propagating COVID-19 transmission, reducing the number of infected children may help decrease the overall spread of the pandemic throughout the population.

This study is subject to several limitations. First, we did not determine the proportion of infections arising from a source outside the household. Changing the level of external exposure of the children, for example through school attendance, would alter the indirect effectiveness of the vaccine (*26*) because parental vaccination would not reduce children’s exposure to infectious nonhousehold members. Second, determination of household membership was based on demographic records in our database. It is possible that some individuals reside at a different location than the address listed or that additional persons (e.g., grandparents or nonparent caregivers) reside in the same household. Third, infections were dated on the basis of the date of sampling, which is invariably several days later than the date of infection. This could result in errors when attributing infections to specific weeks or, in cases where both parent and child became infected, may misclassify the sequence of infections (*27*). Fourth, it is possible that we did not capture important confounders, particularly those related to behavior, which would lead to residual confounding. Fifth, it is possible that outcomes were differentially misclassified between the two study groups—e.g., because a positive diagnosis of an unvaccinated parent would prompt further tests of members of the household. This would result in elevated VE estimates. Lastly, the analysis for secondary attack rate is conditioned on a parent having been infected and on no further infection on days 0 to 2 after the index infection, which are both posttreatment variables. This could result in collider stratification bias.

The results of this study show that parental vaccination confers substantial protection on children residing in the same household. They also shed light on the mechanism through which this protection occurs. These results reinforce the importance of increasing vaccine uptake among the vaccine-eligible population to curb the spread of the SARS-CoV-2 pandemic and protect those who cannot be vaccinated.

## Supplementary Material

20220127-1

## Supplementary Materials

### This PDF file includes:

Materials and Methods Figs. S1 to S3 Tables S1 to S8 References (*29*–*36*)

### Other Supplementary Material for this manuscript includes the following:

MDAR Reproducibility Checklist

## Notes

View/request a protocol for this paper from *Bio-protocol*.
