# Supplementary material for: Indirect protection of children from SARS-CoV-2 infection through parental vaccination
Source: Science. 2022 Jan 27;375(6585):1155–9. doi: 10.1126/science.abm3087 (PMC9799368; doi:10.1126/science.abm3087)
Supplement: 20220127-1 [file science.abm3087.v1.pdf]

Cite as: S. Hayek *et al*., *Science*
10.1126/science.abm3087 (2022).

Indirect protection of children from SARS-CoV-2 infection through parental vaccination

Samah Hayek[1], Galit Shaham[1], Yatir Ben-Shlomo[1], Eldad Kepten[1], Noa Dagan[1,2,3,4], Daniel Nevo[5], Marc Lipsitch[6], Ben Y. Reis[3,4,7], Ran D. Balicer[1,2,8], Noam Barda[2,1,3,4]*

[1]Clalit Research Institute, Clalit Health Services, Ramat Gan, Israel. [2]Software and Information Systems Engineering, Ben Gurion University, Be'er Sheva, Israel. [3]Department of Biomedical Informatics, Harvard Medical School, Boston, MA, USA. [4]The Ivan and Francesca Berkowitz Family Living Laboratory Collaboration at Harvard Medical School and Clalit Research Institute, Boston, MA, USA. [5]Department of Statistics and Operations Research, Tel Aviv University, Tel Aviv, Israel. [6]Center for Communicable Disease Dynamics, Department of Epidemiology, and Department of Immunology and Infectious Diseases, Harvard T.H. Chan School of Public Health, Boston, MA, USA. [7]Predictive Medicine Group, Computational Health Informatics Program, Boston Children's Hospital, Boston, MA, USA. [8]School of Public Health, Faculty of Health Sciences, Ben-Gurion University of the Negev, Beer-Sheba, Israel.

***Corresponding author. Email: noambard@bgu.ac.il**

Children unvaccinated against SARS-CoV-2 may still benefit through protection from vaccinated contacts. We estimated the protection provided to children through parental vaccination with the BNT162b2 vaccine. We studied households without prior infection, consisting of two parents and unvaccinated children, estimating the effect of parental vaccination on the risk of infection for unvaccinated children. We studied two periods separately– an early period (January 17, 2021 - March 28, 2021, Alpha variant, two doses vs. no vaccination) and a late period (July 11, 2021 - September 30, 2021, Delta variant, booster dose vs. two-vaccine doses). We found that having a single vaccinated parent was associated with a 26.0% and 20.8% decreased risk, and having two vaccinated parents was associated with a 71.7% and 58.1% decreased risk, in the early and late periods, respectively. To conclude, parental vaccination confers substantial protection for unvaccinated children in the household.

Since December 2019, SARS-CoV-2 has spread globally (*1*), resulting in over 200 million confirmed infections and over 4 million deaths (*2*). COVID-19 vaccines serve a critical role in combating the spread of the pandemic. Vaccination exerts its effects both through direct protection of vaccinated individuals as well as through indirect protection of individuals living in vaccinated environments (*3*).

Households have specific importance in the context of infectious disease dynamics. Several epidemiological studies have reported that a substantial amount of COVID-19 transmission occurs within settings that include close and prolonged contact, such as households (*4*–*6*). The importance of households in SARS-CoV-2 transmission was highlighted in a recent meta-analysis, in which the secondary attack rate was found to be 19.0% (95% CI: 16.2%, 22.0%) (*7*). The central role of households in SARS-CoV-2 transmission allows them to be used as alternatives to larger clusters for estimating the direct and indirect effects of vaccines (*3*).

Unlike the direct effect of the BNT162b2 mRNA COVID-19 vaccine, which has been extensively explored in clinical trials (*8*) and observational studies (*9*, *10*), the indirect effect of the vaccine has not received as much attention. Previous studies have shown that a single vaccinated household member confers modest protection (42.9%, 95% CI: 22.3%, 58.1%, 10 weeks after the first dose) against SARS-CoV2 infection in other adult unvaccinated household members (*11*). A study from Israel has shown that vaccination reduces the risk of infection and of transmission once an infection is introduced into the household, and that unvaccinated spouses of healthcare workers are protected by their spouse's vaccination (*12*). A different study evaluated the indirect effect at a different level, using 177 geographical communities in Israel, and showed that higher rates of vaccination in each community were associated with a substantial decline in infections among a cohort of unvaccinated individuals aged 16 years or younger (*13*). In general, previous studies concerning indirect effects of vaccination had small sample sizes, included only specific populations (e.g., healthcare workers), did not adjust for certain important confounders, only covered a single period and disease variant and did not explore the mechanism of the indirect effect.

In Israel, the BNT162b2 mRNA COVID-19 vaccine was authorized in December 2020 for individuals aged 16 years and above. In May 2021, this authorization was extended to children and adolescents aged 12 years or older, and in November 2021 to children aged 5 years or older. Third-dose "Booster" shots were initiated in Israel on July 11, 2021, and were gradually extended to cover the entire population - who received the second dose at least 5 months prior - over the month of August. In parallel, from December 2020 to March 2021, Israel underwent a third wave of the COVID-19 pandemic, in which the Alpha variant was dominant. This wave was

accompanied by a nationwide lockdown that included closure of schools and limitation of social activities. A fourth wave occurred in Israel from June to October 2021, this time dominated by the Delta variant. No lockdowns were in effect during this wave, however during the months of July and August the schools were closed for summer vacation. Throughout 2021, COVID-19 PCR tests were freely available nationwide and targeted sampling was performed in schools in which a teacher or a child were found to be infected. In Israel (*14*), as in Europe (*15*) and the US (*16*), the younger age groups remain the least vaccinated.

In this study, we utilize the integrated data repositories of Israel's largest healthcare organization to estimate the indirect Vaccine Effectiveness (VE) of the BNT162b2 mRNA COVID-19 vaccine on unvaccinated children within households. We perform this analysis over two time periods: An early period (January 17, 2021, through March 28, 2021) in children <16 years old, when the Alpha variant was dominant, in which we compare households with parents who were vaccinated with the primary vaccine series to households with unvaccinated parents; and a late period (July 11, 2021, through September 30, 2021) in children <11 years old, when the Delta variant was dominant, in which we compare households with parents who were vaccinated with a booster dose to households in which parents were previously vaccinated with two vaccine doses but have not received the booster dose. In each period we assess the change in the risk of SARS-CoV-2 infection among susceptible children in the household (who are not eligible for vaccination) associated with the vaccination of one or both parents. Furthermore, in each period we explore two of the mechanisms mediating this effect by estimating the decrease in risk that a vaccinated parent would be infected (direct VE), and the decrease in risk that a vaccinated infected parent would then proceed to infect a susceptible child (Household Infectiousness, SAR).

The early period of the study included 400,733 unvaccinated subjects (children and adolescents) from 155,305 distinct households who contributed 2,116,306 person-weeks of follow-up (fig. S1A). The median age of the children was 6 years old (Interquartile Range [IQR]: 3, 9), and 52% of subjects were male. The late period of the study included 181,307 unvaccinated children from 76,621 distinct households who contributed 1,089,191 person-weeks of follow-up (fig. S1B). The median age of the children was 5 years old (IQR: 2, 7), and 52% were male.

Baseline demographic and clinical characteristics of the subjects in each time period are shown in Table 1. A more detailed description, including all potential confounders stratified by parental vaccination status, is presented in table S1. A time-series of the cases observed in our study ("epidemic curve") during both periods, stratified by age group, are presented in fig. S2. Table S2 describes the differences between the infected and uninfected subjects in both study periods.

During the early period, focusing on the Alpha variant and comparing parents vaccinated with the primary vaccine series to unvaccinated parents, a single vaccinated parent was associated with a 26.0% (95% CI: 14.0%, 36.2%) decreased risk of infection for children living in the same household, and two vaccinated parents were associated with a 71.7% (68.6%, 74.6%) decreased risk of infection. This effect was fairly uniform across subject age groups and household sizes. For example, the adjusted VE was 67.1% (52.4%, 77.3%) for a household of size 3 in which both parents were vaccinated, and 62.9% (44.2%, 75.4%) for a household of size 7 in which both parents were vaccinated (Fig. 1 and table S3).

During the late period, focusing on the Delta variant and comparing parents vaccinated with a third (booster) dose to parents who received only two doses at least five months prior, a single boosted parent was associated with a 20.8% (11.4%, 29.1%) decreased risk for infection, while two boosted parents were associated with a 58.1% (53.1%, 62.6%) decreased risk for infection. Some heterogeneity of the effect was observed between age groups and household size. For example, adjusted VE was 65.9% (56.7%, 73.2%) for a subject aged 0-2 years living with two boosted parents, and the adjusted VE was 55.5% (48.6%, 61.6%) for a subject aged 7-11 years living with two boosted parents (Fig. 1 and table S3).

Plots of the predicted versus observed incidence rates indicate a good model fit (fig. S3).

Analysis of the direct effect of the BNT162b2 mRNA COVID-19 vaccine on the risk of parental infection estimated a reduction of 94.4% (93.2%, 95.4%) in the risk of documented infection during the early period (Alpha variant) and 86.3% (83.4%, 88.6%) in the risk of documented infection during the late period (Delta variant) among fully vaccinated adults (Table 2).

Full vaccination of an infected parent was associated with a 72.1% (36.6%, 89.3%) decreased odds of infection of one or more susceptible children in the household from that parent during the early period, and a 79.6% (55.9%, 91.8%) decreased odds of transmission from a boosted, infected parent to one or more susceptible children during the late period (Table 3), in both cases adjusting for the vaccination status of the other parent.

Figure 2 shows a schematic representation of the mechanism by which the direct protection of the parent and the reduction in the secondary attack rate comprise the indirect protection observed for the children.

In the sensitivity analysis using bacterial diarrhea as a negative control outcome, the association ("VE") was -14% (-49%, 13%) for one vaccinated parent and -16% (-37%, 1.8%) for two vaccinated parents (table S4).

In this study, we estimated the indirect protective effect of vaccinating parents with the BNT162B2 mRNA COVID-19

vaccine on their children's risk of SARS-CoV-2 infection in households without prior infection. This estimation was performed both for the primary vaccine series during a period in which Alpha variant was dominant and for the vaccine booster dose during a period in which the Delta variant was dominant. In both periods, we found that parental vaccination substantially reduced the risk of children being infected with SARS-CoV-2, though the effect was somewhat smaller during the late period. While this smaller effect could result from heterogeneity, as the populations are different in composition, it more likely stems from non-boosted parents still being somewhat protected from the first two vaccine doses, which makes the relative effect of the additional booster vaccination dose smaller. Of note, we found the effect for two vaccinated parents to be substantially larger than for a single vaccinated parent in both periods (26.0% => 71.7%, 20.8% => 58.1%). This emphasizes that even a single unvaccinated parent remains an important vector for introducing infections into the household.

Previous findings have also shown a substantial indirect effect for SARS-CoV-2 vaccines. A study focusing on unvaccinated spouses of healthcare workers found the indirect effect to be 43% (23%, 58%) ten weeks after receipt of the first vaccine dose (*11*). A study from Israel found that once COVID-19 is introduced into a household, vaccination reduces infectivity by 78% (30%, 94%) (*12*). Another study from Israel using geographical areas to estimate the community level protection resulting from vaccinated individuals, found that on average, for every 20 percentage points increase in the number of vaccinated individuals, the positive test fraction of the unvaccinated population decreased by a factor of approximately two (*13*). In general, it is difficult to directly compare the findings of these studies with the current study, due to the different designs, adjustments and exposure definitions.

The present study focused on the indirect benefits of vaccinated parents for unvaccinated children. Indirect vaccine effects are mediated by two main mechanisms: First, by protecting potential contacts, vaccination reduces the likelihood that subjects will encounter an infectious individual. Second, vaccination may reduce the infectiousness of vaccinated individuals who do acquire the infection (*17*, *18*). The current study explored these two mechanisms by estimating the direct effect of parental vaccination on parental infection, as well as the vaccination-related change in the risk of infection from an infected parent to a susceptible child. We found the direct effect of parental vaccination with two vaccine doses to be 94.4% (93.2%, 95.4%) for acquiring a documented infection with the Alpha variant, and the direct effect of a booster dose to be 86.3% (83.4%, 88.6%) for acquiring a documented infection with the Delta variant. This high effectiveness when comparing parents who have received the booster dose and those who haven't also hints at waning immunity following the second dose. Furthermore, we found that infectiousness to the children in the household from an infected parent vaccinated with two doses is reduced by 72.1% (36.6%, 89.3%) compared to an unvaccinated parent, and infectiousness from a booster-vaccinated parent is reduced by 79.6% (55.9%, 91.8%) compared to a parent who did not receive the booster vaccination dose, in each case adjusting for the vaccination status of the other parent. It should be emphasized that we should not expect the indirect risk to be equal to the product of the direct risk and the infectiousness, as children may also be infected outside of the household or, potentially, through the other parent. The estimated direct VE of the parents is consistent with previous literature (*9*, *19*), as are the results concerning the reduced SAR (*12*).

To detect possible bias originating from uncontrolled confounding, we performed an analysis using a negative control outcome (NCO) (*20*, *21*), bacterial diarrhea. Bacterial diarrhea was chosen because it plausibly shares confounders (e.g., health-related behavior, hygiene) with the outcome of interest, but should not be affected by the exposure of interest (SARS-CoV-2 vaccine). This analysis did not detect substantial effects, further strengthening our findings and reducing the possibility of meaningful unmeasured confounding.

The protective effect of parental vaccination on children's risk described in this study has particular importance for several reasons: First, while children often experience asymptomatic or mild disease when infected with SARS-CoV-2, some do experience severe disease (*22*, *23*) and enduring post-infection symptoms (known as "long COVID") (*24*), particularly when suffering from some degree of immunosuppression (*25*). Second, because of the important role of households in propagating COVID-19 transmission, reducing the number of infected children may help decrease the overall spread of the pandemic throughout the population.

This study is subject to several limitations. First, we did not determine the proportion of infections arising from a source outside the household. Changing the level of external exposure of the children, for example through school attendance, would alter the indirect effectiveness of the vaccine (*26*), since parental vaccination would not reduce children's exposure to infectious non-household members. Second, determination of household membership was based on demographic records in our database. It is possible that some individuals reside at a different location than the address listed, or that additional family members (e.g., grandparents, non-parent caregivers) reside in the same household. Third, infections were dated based on the date of sampling, which is invariably several days later than the date of infection. This could result in errors when attributing infections to specific weeks or, in cases where both parent and child became infected, may misclassify the sequence of infections (*27*). Fourth, it is possible that we did not capture important

confounders, particularly those related to behavior, which would lead to residual confounding. Fifth, it is possible that outcomes were differentially misclassified between the two study groups, e.g., because a positive diagnosis of an unvaccinated parent would prompt further tests of members of the household. This would result in elevated VE estimates. Lastly, the analysis for secondary attack rate is conditioned on a parent having been infected and on no further infection on days 0-2 following the index infection, which are both post-treatment variables. This could result in collider stratification bias.

In summary, the results of this study show that parental vaccination confers substantial protection to children residing in the same household. They also shed light on the mechanism through which this protection occurs. These results reinforce the importance of increasing vaccine uptake among the vaccine-eligible population to curb the spread of the SARS-CoV-2 pandemic and protect those who cannot be vaccinated.

REFERENCES AND NOTES

1. N. Zhu, D. Zhang, W. Wang, X. Li, B. Yang, J. Song, X. Zhao, B. Huang, W. Shi, R. Lu, P. Niu, F. Zhan, X. Ma, D. Wang, W. Xu, G. Wu, G. F. Gao, W. Tan, China Novel Coronavirus Investigating and Research Team, A Novel Coronavirus from Patients with Pneumonia in China, 2019. *N. Engl. J. Med.* **382**, 727–733 (2020). doi:10.1056/NEJMoa2001017 Medline
2. Center for Systems Science and Engineering (CSSE) at Johns Hopkins University (JHU), "COVID-19 Dashboard" (2022); https://coronavirus.jhu.edu/map.html.
3. M. E. Halloran, M. Haber, I. M. Longini Jr., C. J. Struchiner, Direct and indirect effects in vaccine efficacy and effectiveness. *Am. J. Epidemiol.* **133**, 323–331 (1991). doi:10.1093/oxfordjournals.aje.a115884 Medline
4. Z. Wang, W. Ma, X. Zheng, G. Wu, R. Zhang, Household transmission of SARS-CoV-2. *J. Infect.* **81**, 179–182 (2020). doi:10.1016/j.jinf.2020.03.040 Medline
5. K. Danis, O. Epaulard, T. Bénet, A. Gaymard, S. Campoy, E. Botelho-Nevers, M. Bouscambert-Duchamp, G. Spaccaferri, F. Ader, A. Mailles, Z. Boudalaa, V. Tolsma, J. Berra, S. Vaux, E. Forestier, C. Landelle, E. Fougere, A. Thabuis, P. Berthelot, R. Veil, D. Levy-Bruhl, C. Chidiac, B. Lina, B. Coignard, C. Saura, Investigation Team, Cluster of Coronavirus Disease 2019 (COVID-19) in the French Alps, February 2020. *Clin. Infect. Dis.* **71**, 825–832 (2020). doi:10.1093/cid/ciaa424 Medline
6. I. Dattner, Y. Goldberg, G. Katriel, R. Yaari, N. Gal, Y. Miron, A. Ziv, R. Sheffer, Y. Hamo, A. Huppert, The role of children in the spread of COVID-19: Using household data from Bnei Brak, Israel, to estimate the relative susceptibility and infectivity of children. *PLOS Comput. Biol.* **17**, e1008559 (2021). doi:10.1371/journal.pcbi.1008559 Medline
7. Z. J. Madewell, Y. Yang, I. M. Longini Jr., M. E. Halloran, N. E. Dean, Factors Associated With Household Transmission of SARS-CoV-2: An Updated Systematic Review and Meta-analysis. *JAMA Netw. Open* **4**, e2122240 (2021). doi:10.1001/jamanetworkopen.2021.22240 Medline
8. F. P. Polack, S. J. Thomas, N. Kitchin, J. Absalon, A. Gurtman, S. Lockhart, J. L. Perez, G. Pérez Marc, E. D. Moreira, C. Zerbini, R. Bailey, K. A. Swanson, S. Roychoudhury, K. Koury, P. Li, W. V. Kalina, D. Cooper, R. W. Frenck Jr., L. L. Hammitt, Ö. Türeci, H. Nell, A. Schaefer, S. Ünal, D. B. Tresnan, S. Mather, P. R. Dormitzer, U. Şahin, K. U. Jansen, W. C. Gruber, C4591001 Clinical Trial Group, Safety and Efficacy of the BNT162b2 mRNA Covid-19 Vaccine. *N. Engl. J. Med.* **383**, 2603–2615 (2020). doi:10.1056/NEJMoa2034577 Medline
9. N. Dagan, N. Barda, E. Kepten, O. Miron, S. Perchik, M. A. Katz, M. A. Hernán, M. Lipsitch, B. Reis, R. D. Balicer, BNT162b2 mRNA Covid-19 Vaccine in a Nationwide Mass Vaccination Setting. *N. Engl. J. Med.* **384**, 1412–1423 (2021). doi:10.1056/NEJMoa2101765 Medline
10. E. J. Haas, F. J. Angulo, J. M. McLaughlin, E. Anis, S. R. Singer, F. Khan, N. Brooks, M. Smaja, G. Mircus, K. Pan, J. Southern, D. L. Swerdlow, L. Jodar, Y. Levy, S. Alroy-Preis, Impact and effectiveness of mRNA BNT162b2 vaccine against SARS-CoV-2 infections and COVID-19 cases, hospitalisations, and deaths following a nationwide vaccination campaign in Israel: An observational study using national surveillance data. *Lancet* **397**, 1819–1829 (2021). doi:10.1016/S0140-6736(21)00947-8 Medline
11. J. Salo, M. Hägg, M. Kortelainen, T. Leino, T. Saxell, M. Siikanen, L. Sääksvuori, The indirect effect of mRNA-based Covid-19 vaccination on unvaccinated household members. medRxiv 2021.05.27.21257896 [Preprint] (2021). https://doi.org/10.1101/2021.05.27.21257896.
12. M. Layan, M. Gilboa, T. Gonen, M. Goldenfeld, L. Meltzer, A. Andronico, N. Hozé, S. Cauchemez, G. Regev-Yochay, Impact of BNT162b2 vaccination and isolation on SARS-CoV-2 transmission in Israeli households: An observational study. medRxiv 2021.07.12.21260377 [Preprint] (2021). https://doi.org/10.1101/2021.07.12.21260377.
13. O. Milman, I. Yelin, N. Aharony, R. Katz, E. Herzel, A. Ben-Tov, J. Kuint, S. Gazit, G. Chodick, T. Patalon, R. Kishony, Community-level evidence for SARS-CoV-2 vaccine protection of unvaccinated individuals. *Nat. Med.* **27**, 1367–1369 (2021). doi:10.1038/s41591-021-01407-5 Medline
14. Israel Ministry of Health, "Israel COVID-19 Data Tracker" (2022); https://datadashboard.health.gov.il/COVID-19/general.
15. European Centre for Disease Prevention and Control, "COVID-19 Vaccine Tracker" (2022); https://vaccinetracker.ecdc.europa.eu/public/extensions/Covid-19/vaccine-tracker.html.
16. Mayo Clinic, "U.S. COVID-19 Vaccine Tracker" (2022); www.mayoclinic.org/coronavirus-covid-19/vaccine-tracker.
17. T. J. Vanderweele, E. J. Tchetgen Tchetgen, M. E. Halloran, Components of the indirect effect in vaccine trials: Identification of contagion and infectiousness effects. *Epidemiology* **23**, 751–761 (2012). doi:10.1097/EDE.0b013e31825fb7a0 Medline
18. A. Richterman, E. A. Meyerowitz, M. Cevik, Indirect Protection by Reducing Transmission: Ending the Pandemic with Severe Acute Respiratory Syndrome Coronavirus 2 Vaccination. *Open Forum Infect. Dis.* **9**, ofab259 (2022). doi:10.1093/ofid/ofab259
19. N. Barda, N. Dagan, C. Cohen, M. A. Hernán, M. Lipsitch, I. S. Kohane, B. Y. Reis, R. D. Balicer, Effectiveness of a third dose of the BNT162b2 mRNA COVID-19 vaccine for preventing severe outcomes in Israel: An observational study. *Lancet* **398**, 2093–2100 (2021). doi:10.1016/S0140-6736(21)02249-2 Medline
20. M. Lipsitch, E. Tchetgen Tchetgen, T. Cohen, Negative controls: A tool for detecting confounding and bias in observational studies. *Epidemiology* **21**, 383–388 (2010). doi:10.1097/EDE.0b013e3181d61eeb Medline
21. X. Shi, W. Miao, E. T. Tchetgen, A Selective Review of Negative Control Methods in Epidemiology. *Curr. Epidemiol. Rep.* **7**, 190–202 (2020). doi:10.1007/s40471-020-00243-4 Medline
22. L. S. Shekerdemian, N. R. Mahmood, K. K. Wolfe, B. J. Riggs, C. E. Ross, C. A. McKiernan, S. M. Heidemann, L. C. Kleinman, A. I. Sen, M. W. Hall, M. A. Priestley, J. K. McGuire, K. Boukas, M. P. Sharron, J. P. Burns, International COVID-19 PICU Collaborative, Characteristics and Outcomes of Children With Coronavirus Disease 2019 (COVID-19) Infection Admitted to US and Canadian Pediatric Intensive Care Units. *JAMA Pediatr.* **174**, 868–873 (2020). doi:10.1001/jamapediatrics.2020.1948 Medline
23. J. Toubiana, C. Poirault, A. Corsia, F. Bajolle, J. Fourgeaud, F. Angoulvant, A. Debray, R. Basmaci, E. Salvador, S. Biscardi, P. Frange, M. Chalumeau, J.-L. Casanova, J. F. Cohen, S. Allali, Kawasaki-like multisystem inflammatory syndrome in children during the covid-19 pandemic in Paris, France: Prospective observational study. *BMJ* **369**, m2094 (2020). doi:10.1136/bmj.m2094 Medline
24. J. F. Ludvigsson, Case report and systematic review suggest that children may experience similar long-term effects to adults after clinical COVID-19. *Acta Paediatr.* **110**, 914–921 (2021). doi:10.1111/apa.15673 Medline
25. A. Mastrangelo, W. Morello, E. Vidal, I. Guzzo, L. Annicchiarico Petruzzelli, E. Benetti, M. Materassi, M. Giordano, A. Pasini, C. Corrado, G. Puccio, R. Chimenz, C. Pecoraro, L. Massella, L. Peruzzi, G. Montini, COVID-19 Task Force of the Italian Society of Pediatric Nephrology, Impact of COVID-19 Pandemic in Children with CKD or Immunosuppression. *Clin. J. Am. Soc. Nephrol.* **16**, 449–451 (2021). doi:10.2215/CJN.13120820 Medline

26. E. Goldstein, K. Paur, C. Fraser, E. Kenah, J. Wallinga, M. Lipsitch, Reproductive numbers, epidemic spread and control in a community of households. *Math. Biosci.* **221**, 11–25 (2009). doi:10.1016/j.mbs.2009.06.002 Medline
27. E. K. Accorsi, X. Qiu, E. Rumpler, L. Kennedy-Shaffer, R. Kahn, K. Joshi, E. Goldstein, M. J. Stensrud, R. Niehus, M. Cevik, M. Lipsitch, How to detect and reduce potential sources of biases in studies of SARS-CoV-2 and COVID-19. *Eur. J. Epidemiol.* **36**, 179–196 (2021). doi:10.1007/s10654-021-00727-7 Medline
28. S. Hayek, G. Shaham, Y. Ben-Shlomo, E. Kepten, N. Dagan, D. Nevo, M. Lipsitch, B. Reis, R. Balicer, N. Barda, Indirect protection of children from SARS-CoV-2 infection through parental vaccination, Zenodo (2022); https://doi.org/10.5281/zenodo.5883892.
29. M. A. Hernán, J. M. Robins, Using Big Data to Emulate a Target Trial When a Randomized Trial Is Not Available. *Am. J. Epidemiol.* **183**, 758–764 (2016). doi:10.1093/aje/kwv254 Medline
30. M. E. Halloran, C. J. Struchiner, Study designs for dependent happenings. *Epidemiology* **2**, 331–338 (1991). doi:10.1097/00001648-199109000-00004 Medline
31. M. E. Halloran, The Minicommunity Design to Assess Indirect Effects of Vaccination. *Epidemiol. Methods* **1**, 83–105 (2012). doi:10.1515/2161-962X.1008 Medline
32. L. N. Yelland, A. B. Salter, P. Ryan, Performance of the modified Poisson regression approach for estimating relative risks from clustered prospective data. *Am. J. Epidemiol.* **174**, 984–992 (2011). doi:10.1093/aje/kwr183 Medline
33. G. Y. Zou, A. Donner, Extension of the modified Poisson regression model to prospective studies with correlated binary data. *Stat. Methods Med. Res.* **22**, 661–670 (2013). doi:10.1177/0962280211427759 Medline
34. G. Zou, A modified poisson regression approach to prospective studies with binary data. *Am. J. Epidemiol.* **159**, 702–706 (2004). doi:10.1093/aje/kwh090 Medline
35. D. L. Miglioretti, P. J. Heagerty, Marginal modeling of nonnested multilevel data using standard software. *Am. J. Epidemiol.* **165**, 453–463 (2007). doi:10.1093/aje/kwk020 Medline
36. S. Seaman, M. Pavlou, A. Copas, Review of methods for handling confounding by cluster and informative cluster size in clustered data. *Stat. Med.* **33**, 5371–5387 (2014). doi:10.1002/sim.6277 Medline

ACKNOWLEDGMENTS

Funding: This study was supported by the Ivan and Francesca Berkowitz family living laboratory collaboration at Harvard Medical School and Clalit Research Institute. ML was supported by the Morris-Singer Fund. **Author contributions:** SH, GS, EK, DN, ML and NB conceived and designed the study. GS and YBS performed the data extraction and analysis. All authors wrote the manuscript, critically reviewed it and decided to proceed with publication. RDB and NB supervised the analysis process and vouch for the data and analysis. **Competing interests:** SH, GS, YBS, EK, ND, RDB and NB report institutional grants to Clalit Research Institute from Pfizer outside the submitted work and unrelated to COVID-19, with no direct or indirect personal benefits. ML reports grants from Pfizer, personal fees from Merck, personal fees from Bristol-Meyers Squibb, personal fees from Sanofi Pasteur, personal fees from Janssen, grants from NIH (US), grants from National Institute for Health Research (UK), grants from CDC (US), grants from Open Philanthropy Project, grants from Wellcome Trust, grants from Pfizer outside the submitted work; he has provided unpaid advice on COVID vaccines or vaccine studies to One Day Sooner (nonprofit), CEPI (nonprofit), Pfizer, Astra-Zeneca, Janssen, and COVAXX (United Biosciences). The remaining authors declare no competing interests. **Data and materials availability:** National and organizational data privacy policies prohibit sharing of individual-level data, such as those used for this study, even if anonymized. The analytic code is available at Zenodo (*28*).

This work is licensed under a Creative Commons Attribution 4.0 International (CC BY 4.0) license, which permits unrestricted use, distribution, and reproduction in any medium, provided the original work is properly cited. To view a copy of this license, visit https://creativecommons.org/licenses/by/4.0/. This license does not apply to figures/photos/artwork or other content included in the article that is credited to a third party; obtain authorization from the rights holder before using such material.

SUPPLEMENTARY MATERIALS

science.org/doi/10.1126/science.abm3087
Materials and Methods
Figs. S1 to S3
Tables S1 to S8
References (*29*–*36*)
MDAR Reproducibility Checklist

8 September 2021; accepted 21 January 2022
Published online 27 January 2022
10.1126/science.abm3087

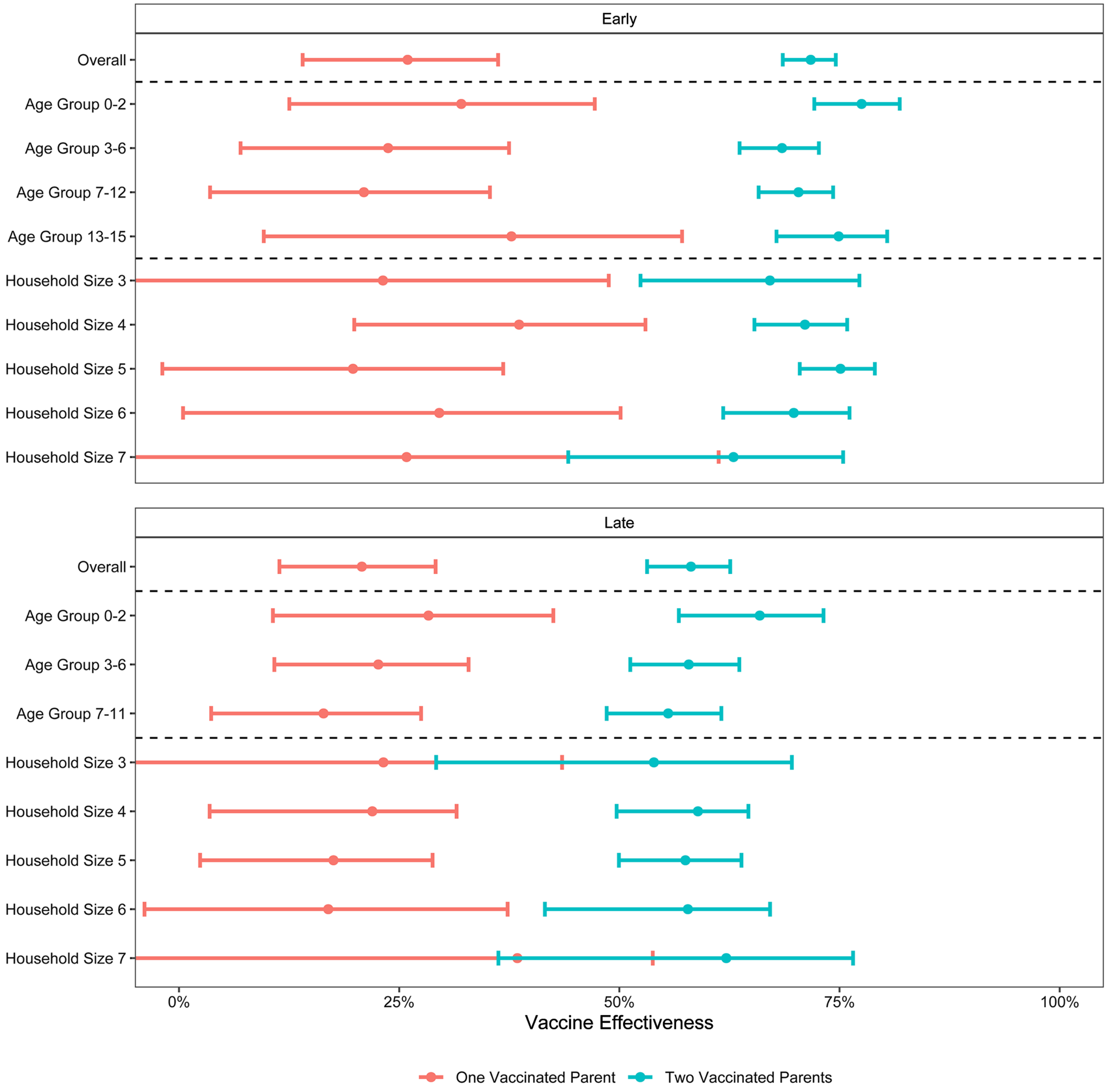

Fig. 1. Indirect Effect of the BNT162B2 mRNA Covid-19 Vaccine by Age Group and Household Size. Indirect vaccine effectiveness (one minus the incidence rate ratio) of one vaccinated parent and two vaccinated parents on the probability of infection of a susceptible child within the household, overall and within age-group and household size categories. Points represent the point estimates, and error bars represent the 95% confidence intervals. The top part shows the first study period (vaccination with two doses at least 7 days prior vs. no vaccination, Alpha variant) and the bottom part shows the second study period (receipt of the booster dose vs. no receipt of the booster dose, Delta variant). The numeric results included in this figure are presented in table S3.

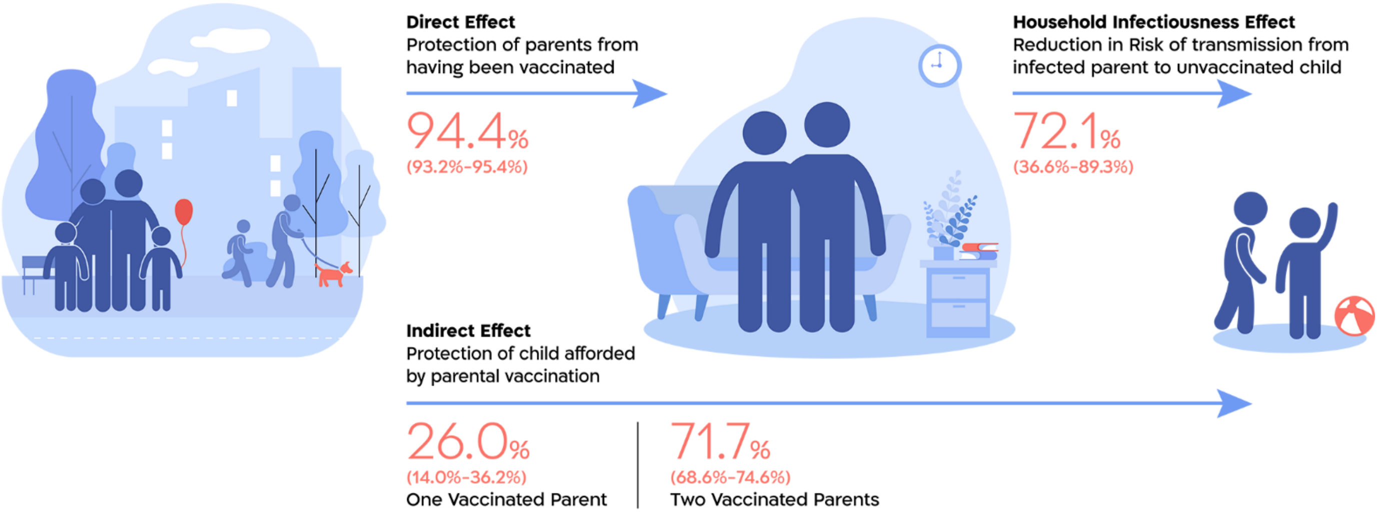

Fig. 2. Mechanism of Disease Transmission. An illustration showing the indirect effect of parental vaccination on children's risk of SARS-CoV-2 infection, and two of its composite parts: The direct effect of vaccination on the parents (estimated as the incidence rate ratio of parental infection between vaccinated and unvaccinated parents) and the risk of transmission from an infected parent to his or her children (estimated as the odds ratio of an infected parent infecting at least one child in the household). Note that we do not expect the indirect risk to equal the product of the direct risk and infectiousness, as children may also be infected outside of the household or, potentially, through the other parent. Estimates shown are from the early study period – in which parents vaccinated with two doses at least 7 days prior were compared to unvaccinated parents, and the dominant variant was Alpha.

Table 1. Descriptive Statistics of the Study Population. The study population includes susceptible children under the age of vaccination eligibility, residing in the households included in the study. The early period was January 17, 2021 to March 28, 2021. The late period was July 11, 2021 to September 30, 2021.

Characteristic	Early Period (N=400,733)	Late Period (N=181,307)
Age (median, IQR)	6 (3-9)	5 (2,7)
Age (N, %)		
0-2	81,672 (20%)	47,710 (26%)
3-6	135,230 (34%)	75,278 (42%)
7-12*	149,917 (37%)	58,319 (32%)
13-15	33,914 (8.5%)	NA
Sex (N, %)		
Female	194,272 (48%)	87,913 (48%)
Male	206,461 (52%)	93,394 (52%)
Population group (N, %)		
Arabs	101,557 (25%)	32,484 (18%)
General	277,444 (69%)	140,222 (77%)
Ultra-Orthodox Jewish	21,732 (5.4%)	8,601 (4.7%)
Socioeconomic Status (N, %)		
Low	223,108 (56%)	88,023 (49%)
Medium	162,833 (41%)	87,380 (48%)
High	14,792 (3.7%)	5,904 (3.3%)
Household size (median, IQR)	5 (4,6)	5(4,5)
Household size (N, %)		
3	20,127 (5.0%)	11,936 (6.6%)
4	107,549 (27%)	63,866 (35%)
5	157,379 (39%)	73,150 (40%)
6	80,423 (20%)	24,878 (14%)
7	35,255 (8.8%)	7,477 (4.1%)
Residence type (N, %)		
Large City	127,887 (32%)	62,991 (35%)
Small City	149,260 (37%)	67,702 (37%)
Town	76,764 (19%)	28,817 (16%)
Rural	31,555 (7.9%)	13,888 (7.7%)
Kibbutz (Communal Residence)	15,267 (3.8%)	7,909 (4.4%)
Obesity (N, %)	24,780 (6.2%)	9,524 (5.3%)
Cardiovascular conditions (N, %)	1,833 (0.5%)	460 (0.3%)

Pulmonary disease (N, %)	47,823 (12%)	18,779 (10%)
Type 2 diabetes (N, %)	1,833 (0.5%)	636 (0.4%)
Hypertension (N, %)	619 (0.2%)	238 (0.1%)
Active malignancy (N, %)	240 (<0.1%)	135 (<0.1%)

*The late period includes children up to age 11.

Table 2. Direct Effect of BNT162B2 mRNA COVID-19 Vaccine. Direct vaccine effectiveness is the reduction in the probability of infection of a fully vaccinated parent compared to an unvaccinated/un-boosted parent, defined as one minus the incidence rate ratio. During the early period, full vaccination was defined as the receipt of two doses at least 7 days prior (compared to no vaccination) and the dominant variant was Alpha. During the late period, full vaccination was defined as receipt of a third dose at least 7 days prior (compared to receipt of only two doses at least 5 months prior) and the dominant variant was Delta. Analysis was performed as per the main analysis, this time using parental infection as the outcome. The model was adjusted for individual and household level characteristics. See table S1 for the full list.

	Early period	Late period
Direct Vaccine Effectiveness<br>(95% CI)	94.4%<br>(93.2%, 95.4%)	86.3%<br>(83.4%, 88.6%)

Table 3. Secondary Transmission Risk. The secondary attack rate (SAR) from an infected parent to susceptible children in the household by parent vaccination status. The unit of observation for this analysis consisted of households in which a parent (the index parent) was infected with SARS-CoV-2. The exposure was the vaccination status of the index parent. The outcome was infection of at least one child in the household at days 3-8 following diagnosis of the index parent. To maintain a well-defined point-of-entry of the infection, we excluded households in which the parent than is not the index parent or a child was diagnosed on days 0-2 following diagnosis of the index parent. During the early period, full vaccination was defined as the receipt of two doses at least 7 days prior (compared to no vaccination) and the dominant variant was Alpha. During the late period, full vaccination was defined as receipt of a third dose at least 7 days prior (compared to receipt of only two doses at least 5 months prior) and the dominant variant was Delta. The adjusted estimate was derived from a logistic regression model adjusted for all the household level characteristics and the vaccination status of the non-index parent.

	Early period	Late period
SAR – Vaccinated Parent (%)	9.0%	9.3%
SAR – Unvaccinated/Un-boosted Parents (%)	24.7%	31.1%
1-Adjusted Odds Ratio (95% CI)	72.1%	79.6%
	(36.6%, 89.3%)	(55.9%, 91.8%)
