# Supplementary material for: Indirect protection of children from SARS-CoV-2 infection through parental vaccination
Source: Science. 2022 Jan 27;375(6585):1155–9. doi: 10.1126/science.abm3087 (PMC9799368; doi:10.1126/science.abm3087)
Supplement: Supplementary file 2 — Materials and Methods Figs. S1 to S3 Tables S1 to S8 References (29–36) [file science.abm3087_sm.pdf]

Supplementary Materials for

Indirect protection of children from SARS-CoV-2 infection through parental vaccination

Samah Hayek *et al*.

Corresponding author: Noam Barda, noambard@bgu.ac.il

Science **375**, 1155 (2022)
DOI: 10.1126/science.abm3087

The PDF file includes:

Materials and Methods
Figs. S1 to S3
Tables S1 to S8
References

Other Supplementary Material for this manuscript includes the following:

MDAR Reproducibility Checklist

Methods

Data

We utilized data from Clalit Health Services (CHS), the largest of four integrated payer-provider healthcare organizations in Israel. CHS maintains an electronic health record (EHR) database with over 4.7 million members, accounting for approximately 52% of the Israeli population, which includes extensive demographic and clinical data. Information in the database allows identification of family relationships (such as parents, spouses, offspring and siblings) and home addresses. The database is linked with data from the Israeli Ministry of Health regarding COVID-19, including PCR-test results, diagnoses, hospitalizations, illness severity determinations and deaths.

Study Design

We conducted a retrospective cohort study aiming to emulate a cluster-randomized mini-community target trial (29), in which households (the clusters of the study) are randomized to have zero, one, or two vaccinated parents. The target trial framework facilitates valid causal inference by guiding the design of observational studies to be analogous to a conceptual randomized clinical trial, which they try to emulate. In this mini-community design, households are considered high-risk settings, where contact between parents and their children results in sufficient transmission to evaluate direct and indirect effects (30,31). Specifically, we assessed the indirect protection of the vaccine by estimating the effect of the number of fully vaccinated parents (zero, one or

two fully vaccinated parents) on the risk of SARS-CoV-2 infection of unvaccinated children in the same household.

The study was performed in two different periods, early and late. The early period was from January 17, 2021, through March 28, 2021, when the dominant strain in Israel was the Alpha (B.1.1.7) variant. During this period, the primary vaccine series of two doses was being administered to individuals aged 16 or older. The late period was from July 11, 2021, through September 30, 2021, when the dominant strain in Israel was the Delta (B.1.617.2) variant. During this period, persons aged 12 or older were eligible for the vaccine and the third (booster) dose was being administered to individuals who had received the second dose at least five months prior.

We included households composed of two spouses listed at the same address, both aged 25-80 years old, along with their children under the minimal age of eligibility for vaccination. All household members needed to have at least one year of continuous membership prior to the study period, except for children younger than 2 years (as CHS membership of new-borns sometimes takes longer to register). We excluded households with no children and households with more than seven members (due to sparse data). Lastly, we excluded households with one or more children eligible to be vaccinated, that is, houses with children aged 16 years or older during the early period or children aged 12 years or older during the late period. Within included households, we excluded person-weeks in which the household included a vaccinated child (some immunosuppressed children under the age of eligibility were still vaccinated), any of the household members died of unrelated causes or any household member was considered

recovered (at least 10 days after a positive test if there were no symptoms for at least 3 days) or partially vaccinated (between the first dose and until 7 days after the second dose). In the late period we also excluded person-weeks of households if either parent was less than 5 months after the second vaccine dose (i.e., ineligible for the booster dose) or less than 7 days after the third vaccine dose.

In both periods, individuals were followed until the earliest of the following events: (1) SARS-CoV-2 infection, (2) death or (3) the end of the study period. The exposure of interest was the number of fully vaccinated parents, represented as a categorical variable: 0, 1 or 2. For the first period, full vaccination was defined as receipt of two vaccine doses with the second dose at least 7 days prior, and fully vaccinated parents were compared to unvaccinated parents. For the second period, full vaccination was defined as receipt of a booster (third) dose at least 7 days prior, and booster-vaccinated parents were compared to parents who had received two doses at least 5 months prior, but had not received the booster. The outcome of interest was RT-PCR confirmed SARS-CoV-2 infection. The timing of the outcome was set based on the sampling date.

Potential confounders were defined by domain expertise, and included demographic and clinical variables measured at the level of the individual (e.g., age, sex and background medical conditions) and at the level of the household (e.g., household size, population sector [General Jewish, Arab, Ultra-orthodox Jewish], socioeconomic status, age of other household members, chronic diseases and medical conditions in other household members). We also adjusted for additional variables such as the calendar week of follow-up, place of residence (district and type as defined by the Israeli Central

Bureau of Statistics), and epidemic activity by location. Supplemental Table 5 provides the complete list of variables, and Supplemental Table 6 provides their definitions. For each variable, we used the last recorded measurement prior to the study period, except for SARS-CoV-2 infection status, vaccination status, pregnancy and epidemic activity by location, which were updated weekly.

Statistical Analysis

A row was created in the study table for each child or adolescent for each week. Each row included the values for the subject's different variables for that week (exposure, outcome and confounders).

The effect measure of interest was vaccine effectiveness (VE), defined as one minus the incidence rate ratio (VE=1-IRR) of one or two fully vaccinated parents compared to no fully vaccinated parents (the reference level). To estimate the IRR, we used multi-variable modified Poisson regression (Poisson regression with a binary outcome) (32-34), estimated using generalized estimating equations (GEE) with robust (Sandwich) standard error estimators. Households were used as the clustering variable, and the time unit used was one week. Households were chosen as the level of clustering, since when multiple clusters are nested within one another (e.g., households and children within households), clustering at the top-level accounts for the multilevel structure (35). As suggested by Seaman et al (36), we used an independence working correlation matrix to mitigate informative cluster size bias, which would otherwise be likely given the study design. The models were adjusted for all the confounders mentioned above

(Supplemental Table 5). Subgroup analyses were performed for different household sizes (3-7) and for different age groups.

A complete-case analysis was conducted to deal with missing data, as missing data are rare in the CHS database for the confounders used in this study. To judge model fit, we plotted the rate of observed events against the rate predicted by the model in deciles across household sizes.

Additional Analyses

We sought to further explore the mechanism of the indirect effect by analyzing two parts of the transmission chain separately: First, we estimated the direct effect of the BNT162b2 mRNA COVID-19 vaccine on the vaccinated parents, and second, we estimated the risk of transmission (Household Infectiousness or secondary attack rate, SAR) from an infected parent to a susceptible child.

For the direct effect, we performed the same analysis described above, with the unit of observation set to be each parent, and the outcome set to be an RT-PCR-confirmed SARS-CoV-2 infection among each parent. We further adjusted for pregnancy and smoking status in the index parent and the vaccination status of the other parent. For the SAR, we changed the unit of observation to be an entire household, with the study population consisting of households in which a parent was diagnosed with infection (the "index parent"). The exposure was the vaccination status of the index parent, and the outcome was infection of at least one child in the household in days 3-8 following

diagnosis of the index parent (to account for the incubation period). To keep the point-of-entry of the infection into the household well-defined, we excluded households in which any member other than the index parent was diagnosed with SARS-CoV-2 within 2 days of the index parent. Modeling was performed using multivariable logistic regression, adjusted for the household level characteristics from the previous models, the calendar week, pregnancy status of both parents and the vaccination status of the other parent. The effect measure was one minus the odds ratio (1-OR).

Lastly, to investigate the possibility of unmeasured confounding, we used bacterial diarrhea as a negative control outcome (20,21). By not being affected by SARS-CoV-2 vaccination but plausibly sharing confounders with the outcome of interest, we can use the negative control outcome to detect confounding bias. This analysis was performed in the early period.

All analyses were performed using the R statistical programming language, version 4.0.2. Technical details of the models are presented in Supplemental Table 7. Model output summaries are presented in Supplemental Table 8.

Ethics

The study was approved by the CHS institutional review board, approval number 0052-20-COM2.

Supplemental Figure 1a: Flow chart for Study Participants during the Early Period, January 17, 2021 - March 28, 2021

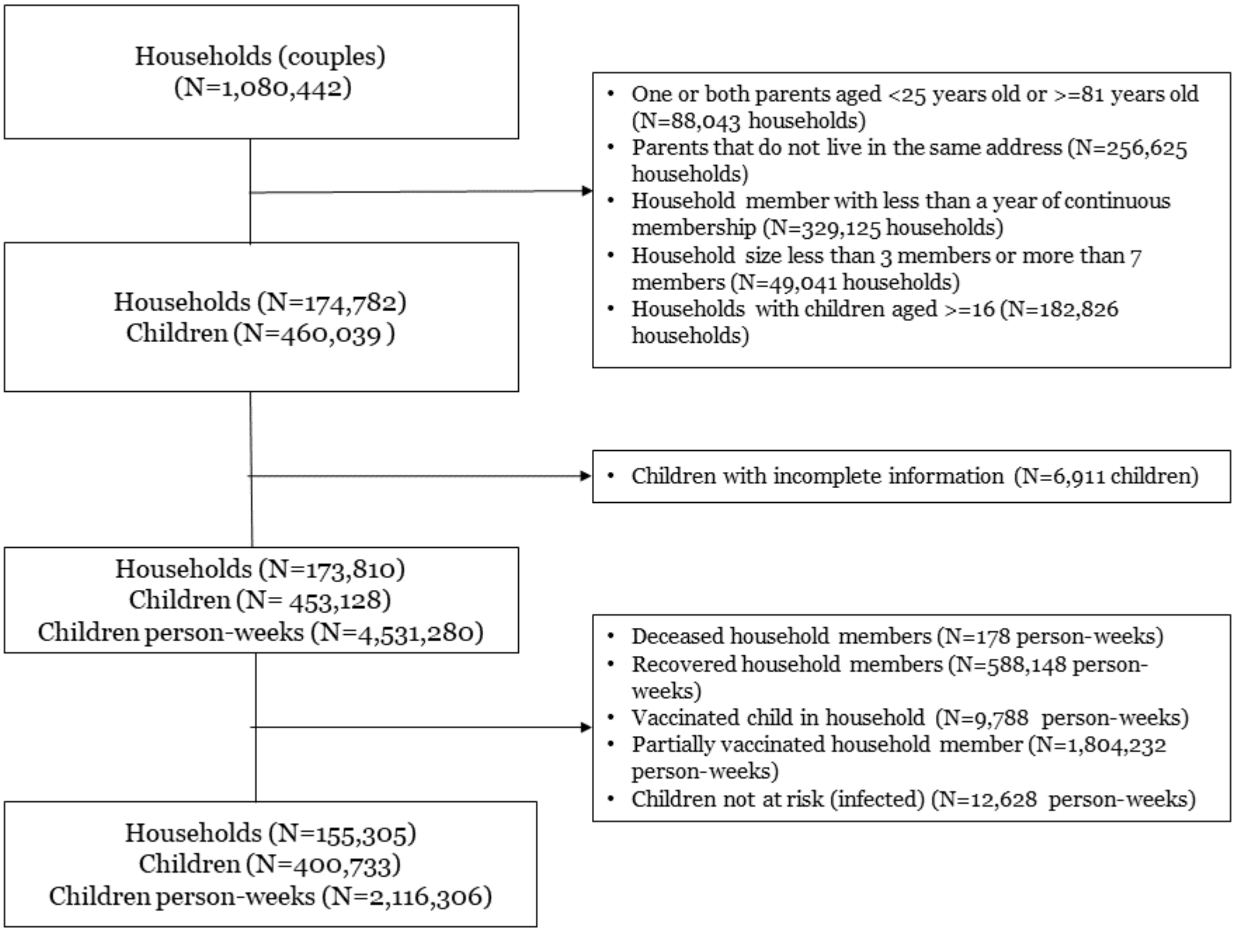

Supplemental Figure 1b: Flow chart for Study Participants During the Late Period, July 11, 2021 - September 30, 2021

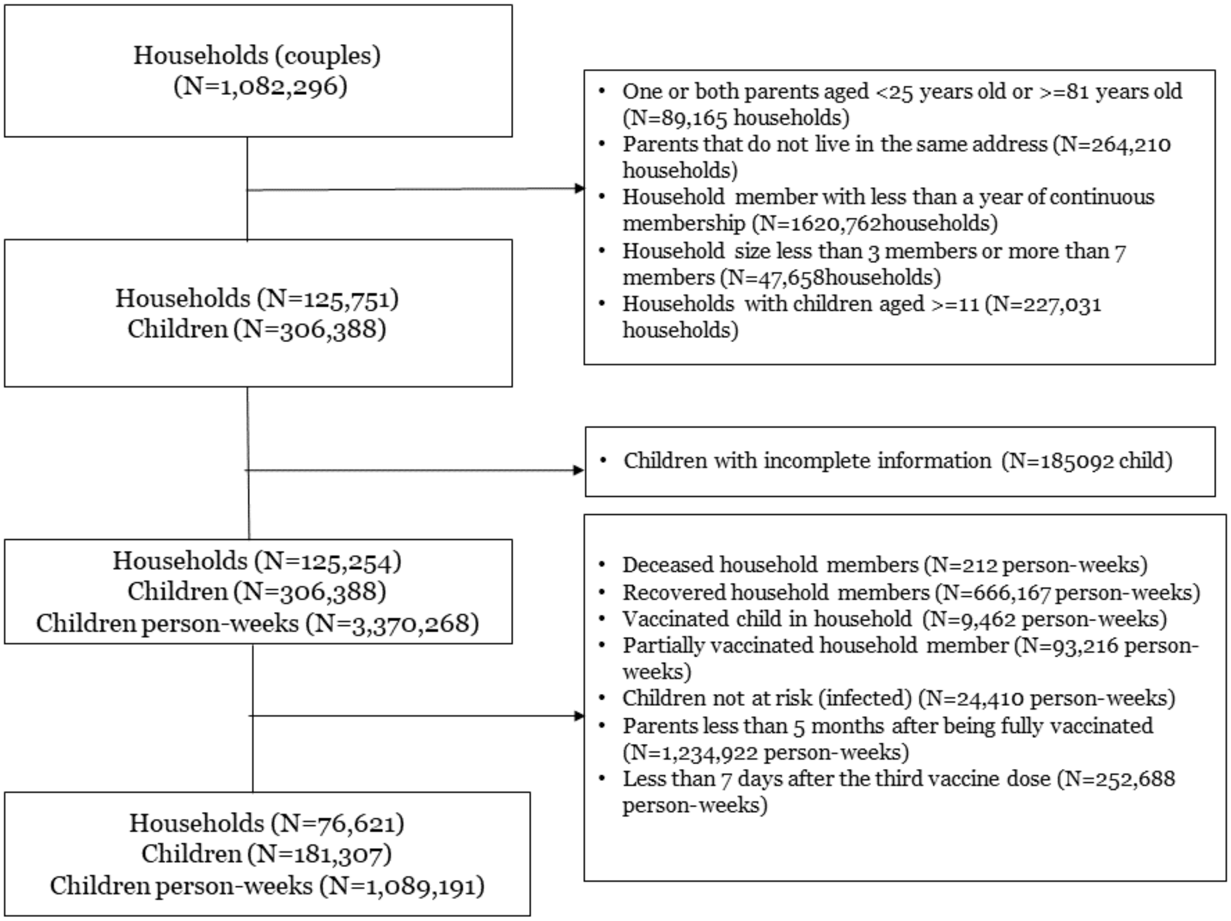

Caption: Study population flow-chart showing the effect of the different inclusion and exclusion criteria on the study population. The arrangement shows selection of households, then children and finally children person-weeks. Panel A shows the early period (January 17, 2021, to March 28, 2021) and panel B shows the late period (July 11, 2021, to September 30, 2021).

Supplemental Figure 2a: Incidence of Infections during the Early Period, by Age Group

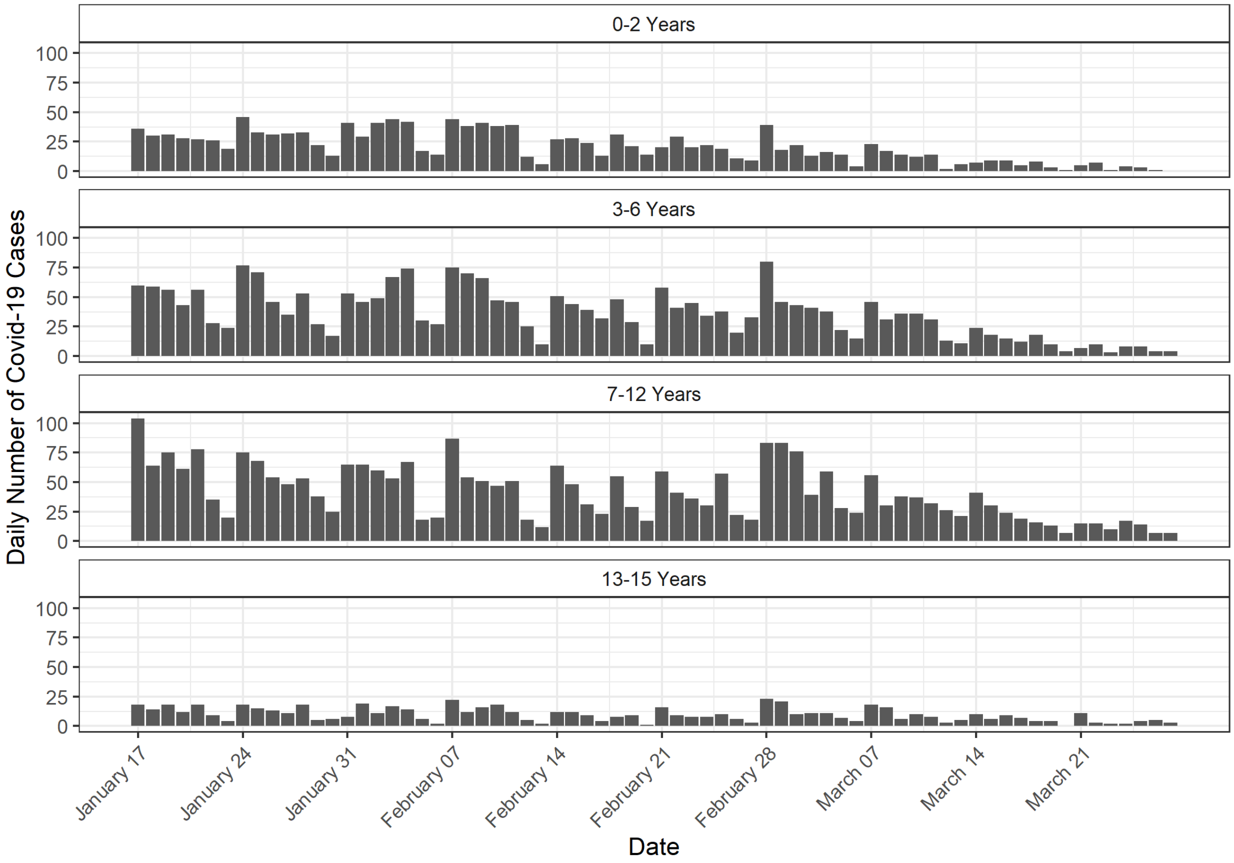

Supplemental Figure 2b: Incidence of Infections during the Late Period, by Age Group

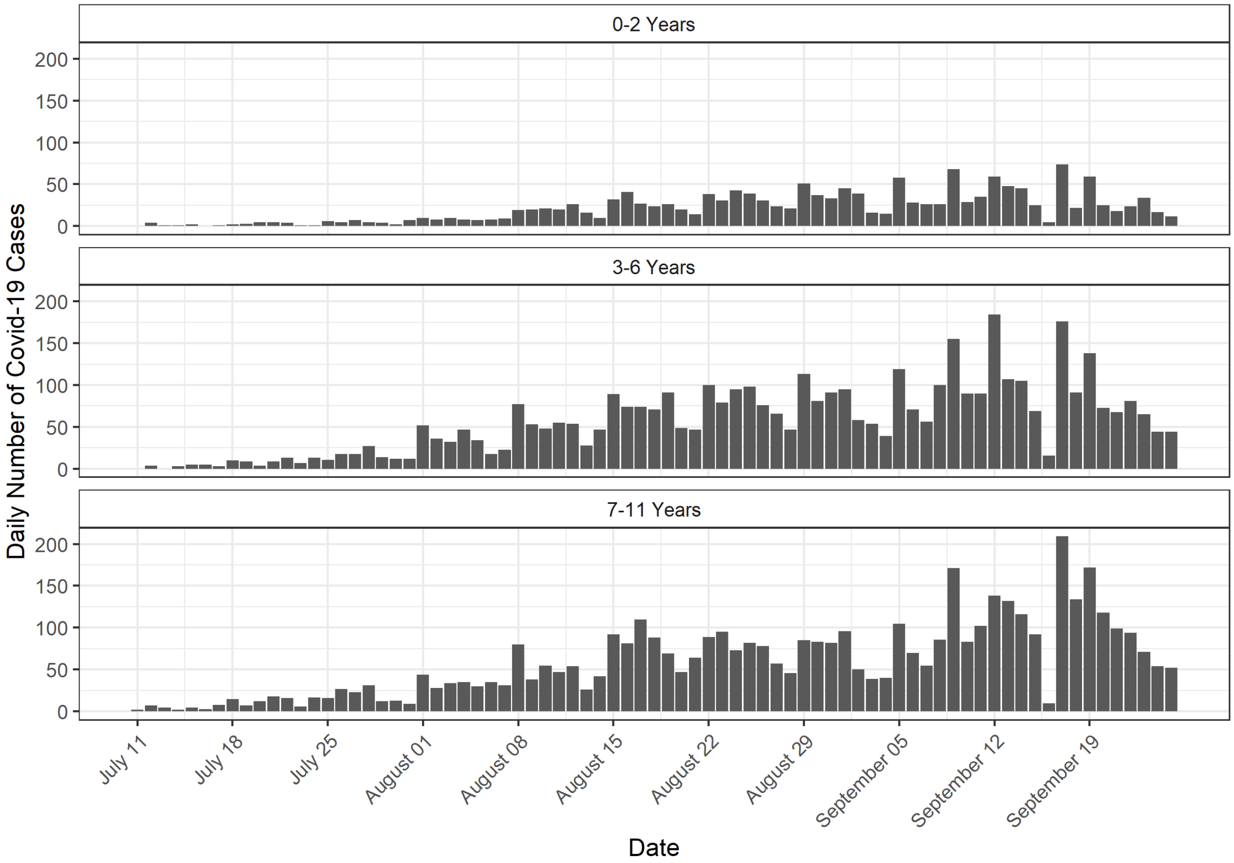

Caption: An “epidemic curve” showing the incidence of cases (RT-PCR confirmed SARS-CoV-2 infections) in the study population (susceptible children under the age of vaccine eligibility) by date. Panel A shows the first study period (vaccination with two doses at least 7 days prior vs. no vaccination, Alpha variant) and panel B shows the second study period (receipt of the booster dose vs. no receipt of the booster dose, Delta variant). The early period was January 17, 2021, to March 28, 2021. The late period was July 11, 2021, to September 30, 2021.

Supplemental Figure 3a: Model Goodness-of-fit for the Early Period

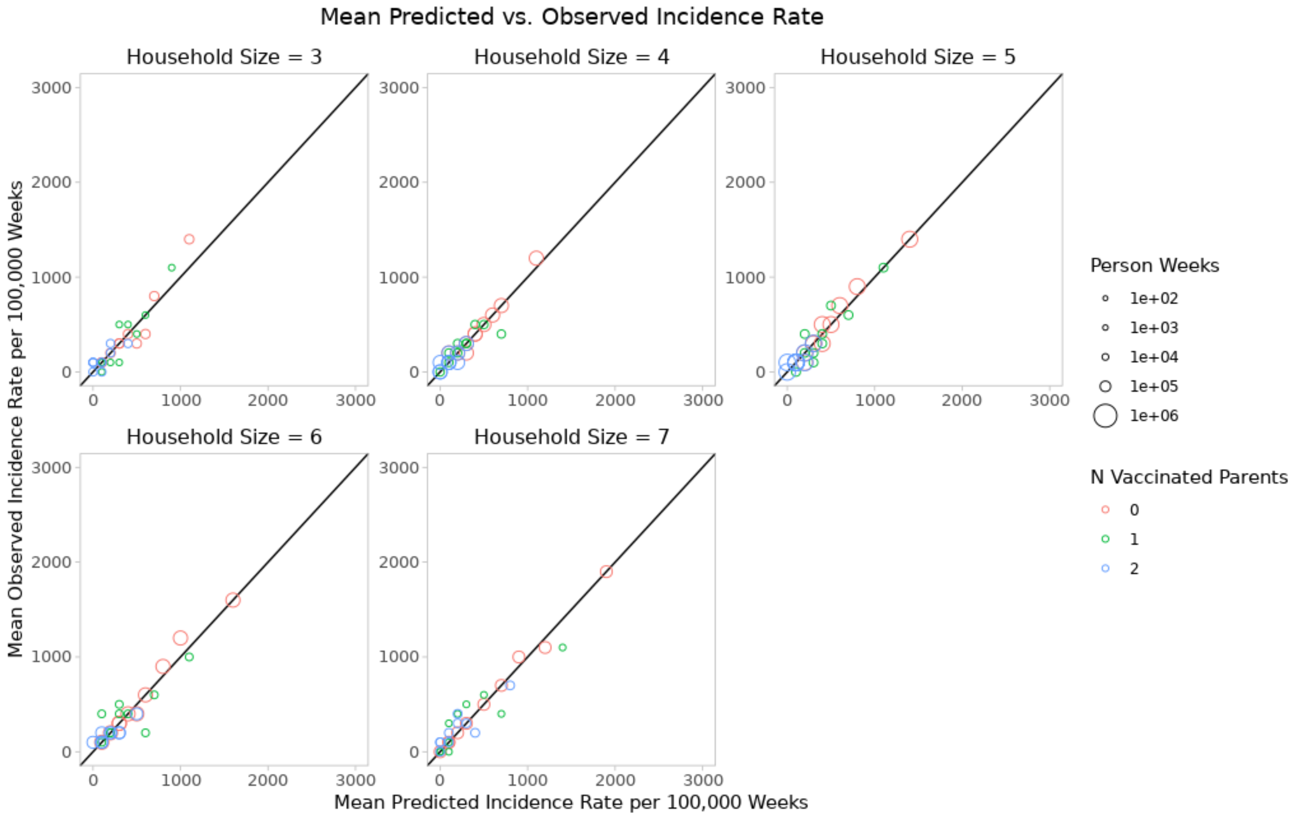

Supplemental Figure 3b: Model Goodness-of-fit for the Late Period

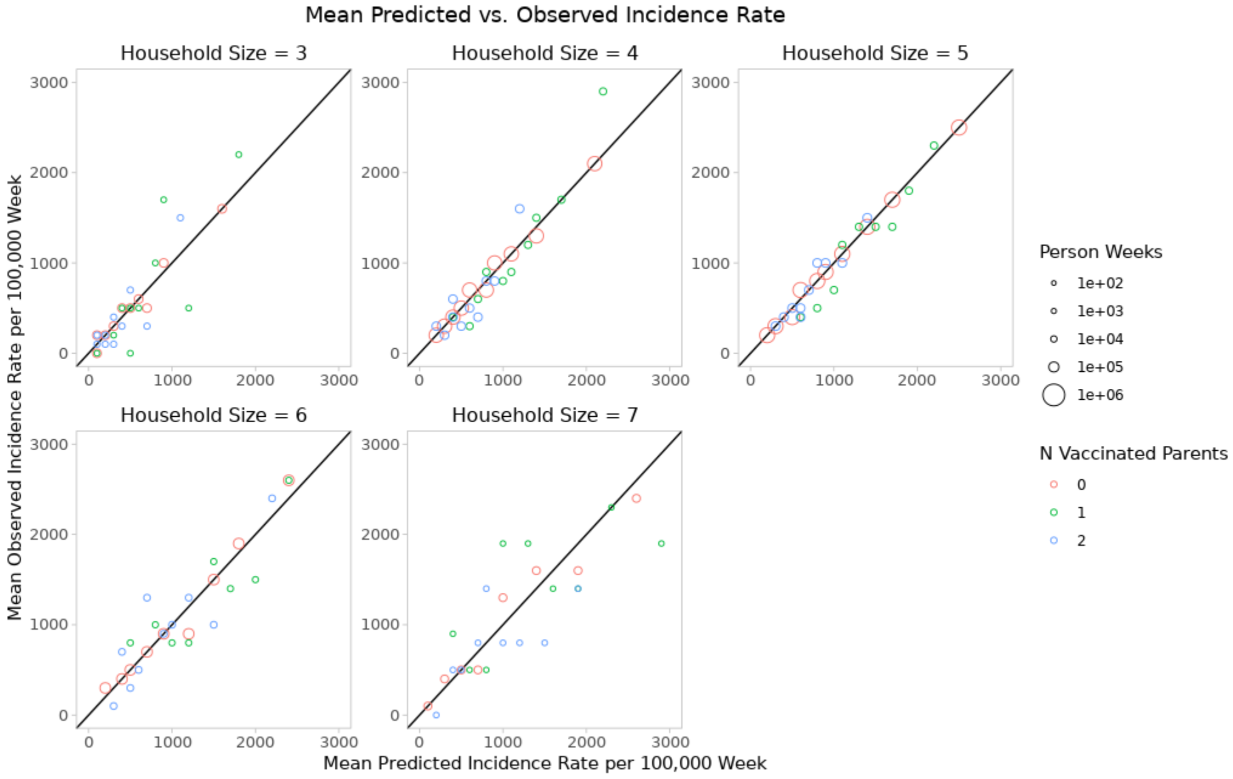

Caption: An observed vs. predicted plot, used to assess the model goodness-of-fit. The Y axis is the mean observed incidence per 100,000 weeks in each decile of predictions. The X axis is the mean predicted incidence per 100,000 weeks in each decile of predictions. The color indicates the exposure (0, 1 or 2 vaccinated parents). The size of each point indicates the number of person-weeks it includes. Values on the diagonal line indicate perfect prediction.

Supplemental Table 1: Descriptive Statistics of the Study Population by Person-weeks, Stratified by Parental Vaccination Status

	Early Period			Late Period		
**Characteristic**	**Unvaccinated parents (1,098,088 Person-weeks)**	**One vaccinated parent (124,228 Person-weeks)**	**Two vaccinated parents (893,990 Person-weeks)**	**Unvaccinated parents (897,361 Person-weeks)**	**One vaccinated parent (58,420 Person-weeks)**	**Two vaccinated parents (133,410 Person-weeks)**
Age (N, %)						
0-2	249,922 (23%)	24,895 (20%)	148,827(17%)	228,366(25%)	14,913 (26%)	31,579(24%)
3-6	392,965 (36%)	42,420 (34%)	277,185(31%)	374,680(42%)	24,101 (41%)	5,373 (40%)
7-12*	380,634 (35%)	45,945 (37%)	372,452(42%)	294,315(33%)	19,406 (33%)	4,809 (36%)
13-15	74,563 (6.8%)	10,967 (8.8%)	95,526(11%)	--	--	--
Sex (N, %)						
Female	568,552 (52%)	64,082 (52%)	459,043(51%)	462,068(51%)	29,995(51%)	68221 (51%)
Male	529,536 (48%)	60,146 (48%)	434,947(49%)	435,293(49%)	28,425(49%)	65189 (49%)
Population group (N, %)						
Arabs	422,747 (38%)	42,353(34%)	108,410(12%)	158,967(18%)	11,032(19%)	6366 (4.8%)

General	590,990 (54%)	77,010(62%)	756,054(85%)	696,696(78%)	44,674(76%)	121944 (91%)
Ultra-Orthodox Jewish	84,351 (7.7%)	4,865(3.9%)	29,526(3.3%)	41,698(4.6%)	2,714 (4.6%)	5100 (3.8%)
Socioeconomic Status (N, %)						
Low	791,714 (72%)	78,606(63%)	332,054(37%)	428, 722(48%)	29,073 (50%)	39,894(30%)
Medium	280,502 (26%)	41,595(33%)	514,383 (58%)	438705(49%)	27,423 (47%)	87,573(66%)
High	25,872 (2.4%)	4,027(3.2%)	47,553(5.3%)	29,934(3.3%)	1,924 (3.3%)	5,943(4.5%)
Household size (median, IQR)	5 (4, 6)	5(4, 6)	5 (4, 5)	5 (4, 5)	5 (4, 5)	5 (4, 5)
Household size (N, %)						
3	59,444 (5.4%)	8,492(6.8%)	35,339(4.0%)	55,172(6.1%)	114 (7.0%)	8,938 (6.7%)
4	260,839 (24%)	36,544 (29%)	252,640(28%)	317,150(35%)	21,631 (37%)	49,105(37%)
5	371,515 (34%)	44,732 (36%)	409,016(46%)	370,824(41%)	22,781 (39%)	56,499 (42%)
6	260,465 (24%)	24,533 (20%)	151,710(17%)	119,083(13%)	7,754(13%)	15,168(11%)
7	145,825 (13%)	9,927 (8.0%)	45,285(5.1%)	35,132(3.9%)	2,140 (3.7%)	3,700(2.8%)
Residence type (N, %)						
Large City	363,629 (33%)	36,524(29%)	272,601(30%)	302,806(34%)	19,267(33%)	50,894(38%)
Small City	415,039 (38%)	48,469 (39%)	324,402 (36%)	335,198 (37%)	22,345(38%)	46,348(35%)
Town	230,254 (21%)	25,935 (21%)	149983 (17%)	145,470 (16%)	9,913(17%)	16,425(12%)
Rural	69,592 (6.3%)	8,969 (7.2%)	87,581 (9.8%)	71,486 (8.0%)	4,563(7.8%)	11,299(8.5%)

Kibbutz (Communal Residence)	19,574 (1.8%)	4,331 (3.5%)	59,423 (6.6%)	42,401 (4.7%)	2,332(4.0%)	8,444(6.3%)
Obesity (N, %)	62,357 (5.7%)	8,160 (6.6%)	58,659 (6.6%)	47,859 (5.3%)	3,151(5.4%)	7,136(5.3%)
Cardiovascular conditions (N, %)	3,440 (0.3%)	352 (0.3%)	2,554 (0.3%)	2,266 (0.3%)	159(0.3%)	349 (0.3%)
Pulmonary disease (N, %)	115,525 (11%)	14,857 (12%)	117,820 (13%)	95,662 (11%)	6,256(11%)	13,446(10%)
Type 2 diabetes (N, %)	5,745 (0.5%)	614 (0.5%)	3,767 (0.4%)	3,254 (0.4%)	196(0.3%)	465(0.3%)
Hypertension (N, %)	1,538 (0.1%)	208 (0.2%)	1,536 (0.2%)	1,168 (0.1%)	79(0.1%)	206(0.2%)
Active malignancy (N, %)	583 (<0.1%)	84 (<0.1%)	572 (<0.1%)	672 (<0.1%)	5 (<0.1%)	111(<0.1%)
Male household members (N, %)						
0	87 (<0.1%)	10 (<0.1%)	117 (<0.1%)	165 (<0.1%)	8 (<0.1%)	38 (<0.1%)
1	308,336 (28%)	40,228 (32%)	274,956 (31%)	311,482 (35%)	20,979 (36%)	48,473 (36%)
2	454,229 (41%)	52,144 (42%)	400,453 (45%)	401,493 (45%)	26,086 (45%)	59,299 (44%)
3	253,469 (23%)	25,214 (20%)	183,019 (20%)	157,014 (17%)	9,329 (16%)	22,270 (17%)
4	72,392 (6.6%)	5,929 (4.8%)	32,391 (3.6%)	25,019 (2.8%)	1,854 (3.2%)	3,060 (2.3%)
5	9,575 (0.9%)	703 (0.6%)	3,054 (0.3%)	2,188 (0.2%)	164 (0.3%)	270 (0.2%)
Adult smokers in the household (N, %)						
0	465,482 (42%)	55,370 (45%)	440,851 (49%)	408,833 (46%)	25,930 (44%)	67,471 (51%)
1	486,885 (44%)	52,263 (42%)	315,850 (35%)	342,542 (38%)	22,974 (39%)	45,179 (34%)

2	145,721 (13%)	16,595 (13%)	137,289 (15%)	145,986 (16%)	9,516 (16%)	20,760 (16%)
Obese household members (N, %)						
0	712,037 (65%)	80,922 (65%)	596,379 (67%)	618,787 (69%)	39,427 (67%)	92,431 (69%)
1	284,721 (26%)	31,164 (25%)	216,702 (24%)	213,258 (24%)	14,477 (25%)	31,041 (23%)
2	80,103 (7.3%)	9,640 (7.8%)	63,092 (7.1%)	54,532 (6.1%)	3,762 (6.4%)	8,313 (6.2%)
3	17,741 (1.6%)	2,005 (1.6%)	14,819 (1.7%)	9,663 (1.1%)	656 (1.1%)	1453 (1.1%)
4	3,123 (0.3%)	387 (0.3%)	2,691 (0.3%)	1,078 (0.1%)	94 (0.2%)	159 (0.1%)
5	344 (<0.1%)	109 (<0.1%)	262 (<0.1%)	43 (<0.1%)	4 (<0.1%)	13 (<0.1%)
6	19 (<0.1%)	1 (<0.1%)	45 (<0.1%)			
Pregnancy in HH (N, %)	104,689 (9.5%)	20,824 (17%)	25,295 (2.8%)	58,284 (6.5%)	5,340 (9.1%)	6,741 (5.1%)
Household members with cardiovascular disease (N, %)						
0	1,044,115 (95%)	117,155 (94%)	842,342 (94%)	858,988 (96%)	55,244 (95%)	12,7161 (95%)
1	52,535 (4.8%)	6,898 (5.6%)	50,590 (5.7%)	37,624 (4.2%)	3,122 (5.3%)	6,106 (4.6%)
2	1,432 (0.1%)	175 (0.1%)	1,054 (0.1%)	735 (<0.1%)	54 (<0.1%)	143 (0.1%)
3	6 (<0.1%)	0 (0%)	4 (<0.1%)	14 (<0.1%)	0 (0%)	0 (0%)
Household members with respiratory disease (N, %)						

0	773,858 (70%)	85,710 (69%)	599,770 (67%)	636,639 (71%)	41,383 (71%)	94,736 (71%)
1	245,625 (22%)	29,319 (24%)	218,713 (24%)	205,014 (23%)	13,566 (23%)	30,762 (23%)
2	61,320 (5.6%)	7,546 (6.1%)	59,858 (6.7%)	46,059 (5.1%)	2,842 (4.9%)	6,627 (5.0%)
3	13,812 (1.3%)	1,421 (1.1%)	13,484 (1.5%)	8,638 (1.0%)	561 (1.0%)	1179 (0.9%)
4	3,073 (0.3%)	214 (0.2%)	1,953 (0.2%)	948 (0.1%)	65 (0.1%)	102 (<0.1%)
5	392 (<0.1%)	18 (<0.1%)	169 (<0.1%)	28 (<0.1%)	3 (<0.1%)	4 (<0.1%)
6	8 (<0.1%)	0 (0%)	43 (<0.1%)	35 (<0.1%)	0 (0%)	0 (0%)
Household members with type 2 diabetes (N, %)						
0	1,035,692 (94%)	115,527 (93%)	842,606 (94%)	858,188 (96%)	55,251 (95%)	126,782 (95%)
1	59,946 (5.5%)	8,330 (6.7%)	49,153 (5.5%)	37,787 (4.2%)	3,046 (5.2%)	6,361 (4.8%)
2	2,388 (0.2%)	365 (0.3%)	2,160 (0.2%)	1377 (0.2%)	123 (0.2%)	267 (0.2%)
3	62 (<0.1%)	6 (<0.1%)	71 (<0.1%)	9 (<0.1%)	0 (0%)	0 (0%)
Household members with hypertension (N, %)						
0	1,011,285 (92%)	110,966 (89%)	789,186 (88%)	822,057 (92%)	52,553 (90%)	119,221 (89%)
1	82,902 (7.5%)	12,663 (10%)	98,585 (11%)	72,120 (8.0%)	5,574 (9.5%)	13,382 (10%)
2	3,754 (0.3%)	594 (0.5%)	6,144 (0.7%)	3,102 (0.3%)	290 (0.5%)	804 (0.6%)
3	139 (<0.1%)	5 (<0.1%)	75 (<0.1%)	70 (<0.1%)	3 (<0.1%)	3 (<0.1%)
4	8 (<0.1%)	0 (0%)	0 (0%)	12 (<0.1%)	0 (0%)	0 (0%)

Household members with active malignancy (N, %)						
0	1,091,083 (99%)	122,746 (99%)	883,268 (99%)	889,718 (99%)	57,847 (99%)	132,064 (99%)
1	6,981 (0.6%)	1,458 (1.2%)	10,660 (1.2%)	7,598 (0.8%)	567 (1.0%)	1330 (1.0%)
2	24 (<0.1%)	24 (<0.1%)	62 (<0.1%)	43 (<0.1%)	6 (<0.1%)	16 (<0.1%)
3				2 (<0.1%)	0 (0%)	0 (0%)
Household members age 0-2 (N, %)						
0	666,050 (61%)	86,234 (69%)	661,257 (74%)	568,652 (63%)	37,505 (64%)	89,228 (67%)
1	377,867 (34%)	34,768 (28%)	217,442 (24%)	302,802 (34%)	19,137 (33%)	41,191 (31%)
2	52,321 (4.8%)	3,134 (2.5%)	15,008 (1.7%)	25,393 (2.8%)	1,690 (2.9%)	2,962 (2.2%)
3	1,800 (0.2%)	87 (<0.1%)	279 (<0.1%)	498 (<0.1%)	78 (0.1%)	29 (<0.1%)
4	50 (<0.1%)	5 (<0.1%)	4 (<0.1%)	16 (<0.1%)	10 (<0.1%)	0 (0%)
Household members age 3-6 (N, %)						
0	457,265 (42%)	59,681 (48%)	449,998 (50%)	362,762 (40%)	24,738 (42%)	57,382 (43%)
1	492,602 (45%)	53,763 (43%)	382,531 (43%)	440,968 (49%)	27,893 (48%)	64,116 (48%)
2	136,165 (12%)	10,136 (8.2%)	58,959 (6.6%)	88,829 (9.9%)	5,451 (9.3%)	11,369 (8.5%)
3	11,802 (1.1%)	631 (0.5%)	2,466 (0.3%)	4,711 (0.5%)	334 (0.6%)	538 (0.4%)
4	254 (<0.1%)	17 (<0.1%)	36 (<0.1%)	91 (<0.1%)	4 (<0.1%)	5 (<0.1%)

Household members age 7-12 (N, %)						
0	477,917 (44%)	53,770 (43%)	332,117 (37%)	456,699 (51%)	30,137 (52%)	64,842 (49%)
1	380,011 (35%)	46,107 (37%)	385,220 (43%)	322,083 (36%)	20,729 (35%)	51,536 (39%)
2	203,765 (19%)	21,487 (17%)	160,507 (18%)	109,635 (12%)	6,989 (12%)	15,915 (12%)
3	35,039 (3.2%)	2,785 (2.2%)	15,708 (1.8%)	8,775 (1.0%)	556 (1.0%)	1,109 (0.8%)
4	1,356 (0.1%)	79 (<0.1%)	438 (<0.1%)	169 (<0.1%)	9 (<0.1%)	8 (<0.1%)
Household members age 13-15 (N, %)						
0	945,205 (86%)	103,484 (83%)	719,934 (81%)	--	--	--
1	125,465 (11%)	17,314 (14%)	152,962 (17%)	--	--	--
2	26,642 (2.4%)	3,361 (2.7%)	20,733 (2.3%)	--	--	--
3	775 (<0.1%)	69 (<0.1%)	361 (<0.1%)	--	--	--
4	1 (<0.1%)	0 (0%)	0 (0%)	--	--	--
Household members age 19-40 (N, %)						
0	93,581 (8.5%)	18,509 (15%)	250,297 (28%)	85,758 (9.6%)	6,007 (10%)	23,884 (18%)
1	204,502 (19%)	29,134 (23%)	211,939 (24%)	162,740 (18%)	13,946 (24%)	30,535 (23%)
2	800,005 (73%)	76,585 (62%)	431,754 (48%)	648,863 (72%)	38,467 (66%)	78,991 (59%)
Household members age 41-60 (N, %)						

0	800,160 (73%)	76,703 (62%)	432,317 (48%)	649,064 (72%)	38,505 (66%)	79,078 (59%)
1	205,192 (19%)	29,251 (24%)	213,425 (24%)	163,026 (18%)	14,027 (24%)	30,724 (23%)
2	92,736 (8.4%)	18,274 (15%)	248,248 (28%)	85,271 (9.5%)	5,888 (10%)	23,608 (18%)
Household members 61-80 (N, %)						
0	1,097,126 (100%)	123,904 (100%)	891,689 (100%)	896,706 (100%)	58,263 (100%)	133,087 (100%)
1	924 (<0.1%)	295 (0.2%)	1,990 (0.2%)	622 (<0.1%)	157 (0.3%)	283 (0.2%)
2	38 (<0.1%)	29 (<0.1%)	311 (<0.1%)	33 (<0.1%)	0 (0%)	40 (<0.1%)
Calendar week for early period (N, %)						
2021-01-17	237,454 (22%)	0 (0%)	0 (0%)	--	--	--
2021-01-24	197,603 (18%)	2,631 (2.1%)	1,790 (0.2%)	--	--	--
2021-01-31	166,398 (15%)	10,042 (8.1%)	14,869 (1.7%)	--	--	--
2021-02-07	135,380 (12%)	15,403 (12%)	45,061 (5.0%)	--	--	--
2021-02-14	98,373 (9.0%)	14,226 (11%)	61,871 (6.9%)	--	--	--
2021-02-21	69,538 (6.3%)	13,161 (11%)	92,818 (10%)	--	--	--
2021-02-28	56,939 (5.2%)	13,853 (11%)	126,738 (14%)	--	--	--
2021-03-07	49,686 (4.5%)	14,799 (12%)	154,850 (17%)	--	--	--
2021-03-14	44,670 (4.1%)	17,311 (14%)	182,094 (20%)	--	--	--
2021-03-21	42,047 (3.8%)	22,802 (18%)	213,899 (24%)	--	--	--

Calendar week for late period (N, %)						
2021-07-11	--	--	--	39,594 (4.4%)	0 (0%)	0 (0%)
2021-07-18	--	--	--	63,203 (7.0%)	0 (0%)	0 (0%)
2021-07-25	--	--	--	84,582 (9.4%)	20 (<0.1%)	0 (0%)
2021-08-01	--	--	--	94,289 (11%)	50 (<0.1%)	0 (0%)
2021-08-08	--	--	--	112,863 (13%)	76 (0.1%)	2 (<0.1%)
2021-08-15	--	--	--	124,176 (14%)	142 (0.2%)	4 (<0.1%)
2021-08-22	--	--	--	105,786 (12%)	295 (0.5%)	29 (<0.1%)
2021-08-29	--	--	--	79,401 (8.8%)	2,870 (4.9%)	3,333 (2.5%)
2021-09-05	--	--	--	72,228 (8.0%)	12,526 (21%)	24,005 (18%)
2021-09-12	--	--	--	64,127 (7.1%)	20,785 (36%)	48,106 (36%)
2021-09-19	--	--	--	57,112 (6.4%)	21,656 (37%)	57,931 (43%)
Epidemic activity by location, ages 0-2 (median, IQR)	0.0056 (0.0041, 0.0072)	0.0055 (0.0042, 0.0070)	0.0052 (0.0037, 0.0066)	0.004 (0.002, 0.009)	0.011 (0.009, 0.013)	0.012 (0.010, 0.013)
Epidemic activity by location, ages 3-6 (median, IQR)	0.0067 (0.0051, 0.0087)	0.0063 (0.0050, 0.0076)	0.0062 (0.0047, 0.0074)	0.008 (0.004, 0.013)	0.018 (0.015, 0.021)	0.019 (0.016, 0.021)
Epidemic activity by location, ages 7-12 (median, IQR)	0.009 (0.007, 0.011)	0.008 (0.006, 0.009)	0.008 (0.006, 0.009)	0.010 (0.005, 0.016)	0.021 (0.017, 0.024)	0.021 (0.018, 0.024)

Epidemic activity by location, ages 13-15 (median, IQR)	0.011 (0.009, 0.013)	0.010 (0.008, 0.012)	0.009 (0.007, 0.011)	0.008 (0.004, 0.012)	0.014 (0.011, 0.016)	0.014 (0.011, 0.016)
Epidemic activity by location, ages 25-64 (median, IQR)	0.014 (0.011, 0.017)	0.011 (0.008, 0.014)	0.009 (0.007, 0.011)	0.007 (0.005, 0.012)	0.012 (0.010, 0.013)	0.011 (0.010, 0.013)
Epidemic activity by location, ages 65-80 (median, IQR)	0.010 (0.007, 0.014)	0.007 (0.005, 0.010)	0.005 (0.004, 0.008)	0.0048 (0.0034, 0.0060)	0.0047 (0.0038, 0.0056)	0.0045 (0.0036, 0.0051)
Minimum months from 2nd dose (N, %)						
5	--	--	--	626,434 (70%)	15,576 (27%)	5,263 (3.9%)
6	--	--	--	248,913 (28%)	30,164 (52%)	57,420 (43%)
7	--	--	--	21,893 (2.4%)	12,525 (21%)	69,438 (52%)
8	--	--	--	121 (<0.1%)	155 (0.3%)	1,289 (1.0%)
Maximum Months from 2nd dose (N, %)						
5	--	--	--	442,102 (49%)	1,889 (3.2%)	908 (0.7%)
6	--	--	--	386,639 (43%)	21,351 (37%)	32,842 (25%)
7	--	--	--	67,415 (7.5%)	33,206 (57%)	92,295 (69%)
8	--	--	--	1,205 (0.1%)	1,974 (3.4%)	7,365 (5.5%)

* The late period includes children up to age 11.

Caption: The study population by person-weeks, as counted in each exposure group, for the two periods. This table includes all variables used in the study. The early period was January 17, 2021, to March 28, 2021. The late period was July 11, 2021, to September 30, 2021.

Supplemental Table 2: Characteristics of Infected and Uninfected Individuals

	Early Period		Late Period	
**Characteristic**	**Infected (N=11,036)**	**Not Infected (N=389,697)**	**Infected (N=13,658)**	**Not Infected (N=167,649)**
Age (median, IQR)	6 (3, 10)	6 (3, 9)	6 (4, 8)	5 (2, 7)
Age Group				
0-2	2,077 (19%)	79,595 (20%)	2,078 (15%)	45,632 (27%)
3-6	3,718 (34%)	131,512 (34%)	5,598 (41%)	69,680 (42%)
7-12*	4,209 (38%)	145,708 (37%)	5,982 (44%)	52,337 (31%)
13-15	1,032 (9.4%)	32,882 (8.4%)	--	--
Sex				
Male	5,598 (51%)	200,863 (52%)	7,098 (52%)	86,296 (51%)
Female	5,438 (49%)	188,834 (48%)	6,560 (48%)	81,353 (49%)
Population Group				
Arab	2,324 (21%)	99,233 (25%)	2,726 (20%)	29,758 (18%)
Ultra-Orthodox Jew	1,361 (12%)	20,371 (5.2%)	943 (6.9%)	7,658 (4.6%)
General	7,351 (67%)	270,093 (69%)	9,989 (73%)	130,233 (78%)
Socioeconomic Status				
Low	7,822 (71%)	215,286 (55%)	7,721 (57%)	80,302 (48%)
Medium	2,962 (27%)	159,871 (41%)	5,596 (41%)	81,784 (49%)
High	252 (2.3%)	14,540 (3.7%)	341 (2.5%)	5,563 (3.3%)
Household size (median, IQR)	5 (4,6)	5 (4,6)	5 (4, 5)	5 (4, 5)
Household Size				

3	462 (4.2%)	19,665 (5.0%)	457 (3.3%)	11,479 (6.8%)
4	2,456 (22%)	105,093 (27%)	4,052 (30%)	59,814 (36%)
5	3,881 (35%)	153,498 (39%)	5,792 (42%)	67,358 (40%)
6	2,773 (25%)	77,650 (20%)	2,406 (18%)	22,472 (13%)
7	1,464 (13%)	33,791 (8.7%)	951 (7.0%)	6,526 (3.9%)
City Type				
Large City	4,022 (36%)	123,865 (32%)	4,596 (34%)	58,395 (35%)
Small City	4,451 (40%)	144,809 (37%)	5,504 (40%)	62,198 (37%)
Town	1,833 (17%)	74,931 (19%)	2,285 (17%)	26,532 (16%)
Rural	624 (5.7%)	30,931 (7.9%)	832 (6.1%)	13,056 (7.8%)
Kibbutz (Communal Residence)	106 (1.0%)	15,161 (3.9%)	441 (3.2%)	7,468 (4.5%)
Obesity	797 (7.2%)	23,983 (6.2%)	906 (6.6%)	8,618 (5.1%)
Type 2 Diabetes	54 (0.5%)	1,779 (0.5%)	48 (0.4%)	588 (0.4%)
Hypertension	23 (0.2%)	596 (0.2%)	18 (0.1%)	220 (0.1%)
Cardiovascular Conditions	33 (0.3%)	1,152 (0.3%)	31 (0.2%)	429 (0.3%)
Pulmonary Disease	1,528 (14%)	46,295 (12%)	1,711 (13%)	17,068 (10%)
Active Malignancy	11 (<0.1%)	229 (<0.1%)	8 (<0.1%)	127 (<0.1%)

* The late period included children up to age 11

Caption: Characteristics of children who did and did not experience the outcome (RT-PCR positive SARS-CoV-2 infection) at each time period. The early period was January 17, 2021, to March 28, 2021. The late period was July 11, 2021, to September 30, 2021.

Supplemental Table 3: Indirect effect of BNT162B2 mRNA COVID-19 Vaccine by Age Group and Household Size

	Early period		Late period	
**Characteristic**	**One Vaccinated Parent**	**Two Vaccinated Parents**	**One Vaccinated Parent**	**Two Vaccinated Parents**
Overall	26.0% (14.0%, 36.2%)	71.7% (68.6%, 74.6%)	20.8% (11.4%, 29.1%)	58.1% (53.1%, 62.6%)
Age Group				
0-2	32.0% (12.5%, 47.2%)	77.50% (72.1%, 81.8%)	28.3% (10.7%, 42.5%)	65.9% (56.7%, 73.2%)
3-6	23.7% (7.0%, 37.5%)	68.5% (63.6%, 72.7%)	22.6% (10.8%, 32.8%)	57.9% (51.2%, 63.6%)
7-12*	21.0% (3.5%, 35.3%)	70.3% (65.8%, 74.3%)	16.4% (3.6%, 27.5%)	55.5% (48.6%, 61.6%)
13-15	37.7% (9.6%, 57.1%)	74.9% (67.8%, 80.4%)	--	--
Household Size				
3	23.2% (-15.9%, 48.8%)	67.1% (52.4%, 77.3%)	23.2% (-17.9%, 50.0%)	53.9% (29.4%, 69.9%)
4	38.6% (19.9%, 52.9%)	71.1% (65.3%, 75.9%)	21.9% (6.6%, 34.8%)	58.9% (50.8%, 65.6%)
5	19.8% (-1.9%, 36.8%)	75.1% (70.5%, 79.0%)	17.5% (1.9%, 30.7%)	57.5% (49.5%, 64.3%)
6	29.6% (0.45%, 50.1%)	69.8% (61.8%, 76.1%)	16.9% (-11.4%, 38.1%)	54.8% (37.8%, 67.2%)
7	25.8% (-42.0%, 61.3%)	62.9% (44.2%, 75.40%)	38.4% (-14.2%, 66.8%)	62.1% (35.3%, 77.8%)

* The late period included children up to age 11

Caption: The indirect effect of parental vaccination on children's risk of SARS-CoV-2 infection. Vaccine effectiveness is defined as one minus the incidence rate ratio comparing one or two fully vaccinated parents to both parents unvaccinated/un-boosted. The early period was January 17, 2021 to March 28, 2021. The late period was July 11, 2021 to September 30, 2021. During the early period, full vaccination was defined as the receipt of two doses at least 7 days prior (compared to no vaccination) and the dominant variant was Alpha. During the late period, full vaccination was defined as receipt of a third dose at least 7 days prior (compared to receipt of only two doses at least 5 months prior) and the dominant variant was Delta. The numbers in parentheses are 95% confidence intervals. Household sizes include the parents, so for example, a household of size 3 has one child.

Supplemental Table 4: Negative Control Analysis for Bacterial Diarrhea

	One Vaccinated Parent (95% CI)	Two Vaccinated Parents (95% CI)
Vaccine effectiveness (95% CI)	-14% (-49%, 13%)	-16% (-37%, 1.8%)

Caption: The reduction in the probability of a child being diagnosed with bacterial diarrhea in a household with one or two fully vaccinated parents compared to a household with no fully vaccinated parents. Bacterial diarrhea is not affected by SARS-CoV-2 vaccination, but is susceptible to the same confounders as the main outcome (SARS-CoV-2 infection), making it a negative control outcome. This analysis was performed in the early period.

Supplemental Table 5: Variables Used for Adjustment of Models

Variable	Values	Time
Individual Level Variables		
Sex	Male, Female	Last before study
Age	Early Period: 0-2, 3-6, 7-12, 13-15<br>Late Period: 0-2, 3-6, 7-11	Last before study
Obesity	Yes, No	Last before study
Active malignancy	Yes, No	Last before study
Type 2 diabetes	Yes, No	Last before study
Cardiovascular disease	Yes, No	Last before study
Hypertension	Yes, No	Last before study
Respiratory disease	Yes, No	Last before study
Smoking	Yes, No	Last before study
Pregnancy in index member	Yes/No	Weekly
Household Level Variables*		
Parental Vaccination Status**	Yes/No	Weekly
Residence type	Large city, Small City, Town, Rural, Kibbutz	Last before study
District	Center, Dan-Petach-Tikva, Eylat, Haifa, Jerusalem, North, Sharon-Shomron, South, Tel-Aviv	Last before study
Population sector	General, Arab, Ultra-Orthodox-Jewish	Last before study
Socioeconomic status	Low, Medium, High	Last before study
Household size	Integer	Last before study

Household members age 0-2	Integer	Last before study
Household members age 3-6	Integer	Last before study
Household members age 7-12	Integer	Last before study
Household members age 13-15	Integer	Last before study
Household members age 19-40	Integer	Last before study
Household members age 41-60	Integer	Last before study
Household members age 61-80	Integer	Last before study
Male household members	Integer	Last before study
Adult smokers in the household	Integer	Last before study
Obese household members	Integer	Last before study
Household members with cardiovascular disease	Integer	Last before study
Household members with hypertension	Integer	Last before study
Household members with type 2 diabetes	Integer	Last before study
Household members with respiratory disease	Integer	Last before study
Household members with active malignancy	Integer	Last before study
Minimum months from second dose***	Integer	Weekly
Maximum months from second dose***	Integer	Weekly
Pregnant household member	Yes/No	Weekly
Calendar week for each study period	Categorical	Weekly

Epidemic activity by location, ages 0-2	Proportion	Weekly
Epidemic activity by location, ages 3-6	Proportion	Weekly
Epidemic activity by location, ages 7-12	Proportion	Weekly
Epidemic activity by location, ages 13-15	Proportion	Weekly
Epidemic activity by location, ages 25-64	Proportion	Weekly
Epidemic activity by location, ages 65-80	Proportion	Weekly
Variable Lists		
Model for indirect effectiveness	Number of fully vaccinated parents (the primary exposure); all individual level variables other than pregnancy status and smoking; all household level variables other than months from second vaccine dose	
Models for direct effectiveness	Vaccination of the index parent (the primary exposure); All other variables including vaccination of the other parent	
Models for secondary attack rate	Vaccination of the index infected parent (the primary exposure); vaccination of the other parent; Pregnancy status of the index infected parent; All household level variables excluding epidemic activity	

* In the models for indirect and direct vaccine effectiveness, “household members” refers to the members of the household other than the one currently being observed. In the model for secondary attack rate, it is the overall number among all household members.

** In the model for indirect VE, the number of fully vaccinated parents is the primary exposure. In the model for direct VE, the primary exposure is the vaccination status of the parent currently being observed, and model is adjusted for the vaccination status of the other parent. In the model for secondary attack rate, the primary exposure is the vaccination status of the index infected parent, and model is adjusted for the vaccination status of the other parent.

*** Time from second dose variables were only used in the late period.

Legend: Variables used for adjustment of the different multivariable models in the study, and the time in which they were extracted.

Supplemental Table 6: Variable Definitions

Diagnosis	Definition
Vaccination status	Fully vaccinated, early period: 2nd dose + 7 days<br>Fully vaccinated, late period: 3rd dose + 7 days, where the 3rd dose is given at least 5 months after the second dose.<br>Partially vaccinated, early period: From receipt of the 1st dose and until 6 days following receipt of the 2nd dose.<br>Partially vaccinated, second period: During the 6 days from receipt of the 3rd dose, given at least 5 months after the second dose<br>Unvaccinated, early period: did not receive any Covid-19 vaccine<br>Unvaccinated, late period: received 2nd dose at least 5 months prior, but did not receive the 3rd dose
Recovered	As defined by the Israel Ministry of Health, usually as 10 days after the positive test if there were no symptoms for at least 3 days, but there are special cases. The final decision is made by the family physician, who documents it in the health record.
Epidemic activity by location	The proportion of infections among CHS members in a specific age group in this specific week, in the area and city type of this specific person, across the following age groups: 0-2, 3-6, 7-12, 13-15, 25-64, and 65-80.
Calendar week (early period)	2021-01-17, 2021-01-24 2021-01-312021-02-07, 2021-02-14, 2021-02-21, 2021-02-28, 2021-03-07, 2021-03-14, 2021-03-21
Calendar week (late period)	2021-07-11, 2021-07-18, 2021-07-25, 2021-08-01, 2021-08-08, 2021-08-15, 2021-08-22, 2021-08-29, 2021-09-05, 2021-09-12, 2021-09-19
Obesity	For individuals of age ≥19, BMI ≥30 kg/m² was defined as obese and BMI <30 Kg/m² were defined as non-obese.<br>For individuals of age 0-18, individuals in BMI percentile 85% and above were considered obese and individuals in BMI percentiles <85% were considered non-obese.
Type 2 Diabetes Mellitus	One of the following:

1. Plasma glucose ≥ 200 mg/dl ever
2. HbA1C ≥ 7% ever
3. HbA1C ≥ 6.5% and plasma glucose ≥ 126 mg/dl three months before or after the HbA1C measurement
4. ICD9 diagnosis code 250* and (HbA1C ≥ 6.5% or plasma glucose ≥ 126) 12 months before the diagnosis or 3 months after it.
5. Purchase of drug with ATC3 code A10B or A10A and (HbA1C ≥ 6.5% or plasma glucose ≥ 126) 12 months before the purchase or 3 months after it.
6. ICD9 diagnosis code 250* and purchase of drug with ATC3 code A10B or A10A 12 months before the diagnosis or 3 months after it.

Hypertension	ICD-9 Diagnosis codes: 401*; 402*; 403*; 404*; 405*<br>ICPC code: K86
Active Malignancy	Defined as a diagnosis with any cancer in the year prior to the index date<br><br>ICD-9 codes: 140-209, V10<br><br>Internal Billing Codes: Orthopedic Oncologic Operations, External Radiotherapy, Brachytherapy, Bone Marrow Transplant, I-131 Treatment, Oncologic Day Hospitalization, In-Hospitalization Chemotherapy, Post-Oncologic Excision Breast Reconstruction, Urinary Bladder Irrigation w/ Chemotherapy, Somatic FISH Test, RFA of Liver Tumor, Intra-Arterial Chemotherapy, Neck RLND, Radical Prostatectomy, BSO, Whipple Operation<br><br>Drugs: 5-Aminolvulinic Acid, Abiraterone, Afatinib, Alemtuzumab, Altretamine, Aminoglutetimide, Anagrelide, Arsenic Trioxide, Asparaginase, Axitinib, Azacitidine, Bendamustine, Bevacizumab, Bleomycin, Bortezomib, Brentuximab Vedotin, Busulfan, Cabazitaxel, Carboplatin, Carfilzomib, Carmust ine, Ceritinib, Cetuximab, Chlorambucil, Chlormethine, Cisplatin, Cladribine, Clofarabine, Crizotinib, Cyclophosphamide, Cytarabine, Dabrafenib, Dacarbazine, Dactinomycin, Dasatinib, Daunorubicine, Decitabine, Degarelix, Diethylstilbestrol, Docetaxel, Doxorubicin, Doxorubicin Liposomal, Epirubicin, Erlotinib, Estramustine, Etoposide, Everolimus, Fludarabine, Flutamide, Formestane, Fotemustine, Fulvestrant,

	Gefitinib, Gemcitabine, Gemtuzumab Ozogamicin, Ibrutinib, Idarubicin, Ifosfamide, Imatinib, Ipilimumab, Irinotecan, Lapatinib, Leuprorelin, Lomustine, Medroxyprogesterone, Megestrol, Melphalan, Methotrexate, Miltefosine, Mitomycin, Mitotane, Mitoxantrone, Nelarabine, Nilotinib, Obinutuzumab, Oxaliplatin, Paclitaxel, Panitumumab, Pazopanib, Pegaspargase, Pemetrexed, Pentostatin, Pertuzumab, Ponatinib, Procarbazine, Regorafenib, Rituximab, Romidepsin, Ruxolitinib, Sorafenib, Streptozocin, Sunitinib, Tegafur, Temozolomide, Temsirolimus, Testolactone, Thiotepa, Tioguanine, Topotecan, Trabectedin, Trastuzumab, Trastuzumab Emtansine, Tretinoin, Tegafur/uracil, Vandetanib, Vemurafenib, Vinblastine, Vincristine, Vinorelbine, Visemodegib
Cardiovascular disease	Having at least one diagnosis of the following:<br><u>Acute, but ill-defined CVD:</u><br>ICD-9 codes: 436*; 437.1; 437.9<br><u>Ischemic Stroke:</u><br>ICD-9 codes: 433; 433._; 433._1; 434*; 362.31; 362.32; 362.33<br>ICPC code: K90<br><u>Hemorrhagic Stroke:</u><br>ICD-9 codes: 430*; 431*<br><u>Transient Ischemic attack:</u><br>ICD-9 codes: 435*; 362.3*<br><u>Coronary heart disease:</u><br>ICD-9 codes: 410*; 411*; 413*; 414*<br><u>Peripheral vascular disease:</u><br>ICD-9 codes: 443*; 440.2*; 440.3*; 440.4*; 440.9*; 250.7*; 445.0*; 443.9<br><u>Heart failure:</u><br>ICD-9 codes: 428*<br><u>Procedure Codes:</u><br>ICD-9 codes: 36.0*; 36.1*
Pulmonary Disease	One or more of:<br><u>COPD</u><br>ICD-9 codes: 491.2*; 492; 492.0; 492.8; 496*<br>ICPC code: R95<br><u>Asthma</u>

ICD-9 code: 493*

<u>Bronchiectasis</u>

ICD-9 codes: 494.0; 494.1

<u>Pulmonary fibrosis</u>

ICD-9 codes: 516.31; 516.30; 516.8; 516.9; 515.0; 501.0

Note: The wildcard * indicates zero or more of any character. The wildcard _ indicates any single character

Supplemental Table 7: Technical Notes

Item	Details
Model for Indirect Effect	Let Y_{ijt} be the infection status of child j in household i in week t (whenever household i contributes follow-up time in week t). Let $A_{it} \in \{0,1,2\}$ be the number of vaccinated parents in household i in week t, and let X_{it} and W_{ijt}, be household- and child-level covariates at time t (some covariates are fixed across t, see Supplemental Table 1). The modified Poisson model (34) is then<br>$$Y_{ijt} \sim Pois(\lambda_{ijt})$$ $$\lambda_{ijt} = E\left[Y_{ijt} \middle\| A_{it}, X_{it}, W_{ijt}\right] = \exp(\beta_0 + \beta_1 I\{A_{it} = 1\} + \beta_2 I\{A_{it} = 2\} + \beta_3^T X_{it} + \beta_4^T W_{ijt})$$<br>Under this model, $\exp(\beta_1)$ is the adjusted relative risk of infection comparing having one vaccinated parent to none and $\exp(\beta_2)$ is the adjusted relative risk of infection comparing having two vaccinated parents to none.<br><br>To estimate the model parameters (the $\boldsymbol{\beta}$'s), the generalized estimating equations approach under independence correlation structure (33,34) was used, with clusters specified as the household. The sandwich estimator for clustered data (33) is used to estimate the standard error.
Model for Direct Effect	Same as the model for the direct effect, with the design modifications specified in the manuscript.
Model for Secondary Attack Rate	Let $\boldsymbol{Y_i}$ be the outcome (whether at least one child in household $\boldsymbol{i}$ was infected on days 3-8 following infection of the index parent), $\boldsymbol{A_i} \in \{\boldsymbol{0}, \boldsymbol{1}\}$ be the vaccination status of the index parent, and $\boldsymbol{X_i}$ the household-level covariates (see Supplemental Table 1). The logistic regression model is then<br>$$Y_i \sim Bernoulli(P_i)$$ $$Logit(P_i) = \beta_1 A_i + \beta_2{}^T X_i$$<br>Under this model, $exp(\beta_1)$ is the adjusted odds ratio of infection, comparing a household in which the index infected parent is vaccinated to a household in which the index infected parent is not vaccinated.<br><br>The model was estimated using standard Maximum likelihood estimation.

Caption: Technical notes regarding the models and estimation techniques used in the study.

Supplemental Table 8: Model Output Summaries

Variable	Estimate	Standard Error	Confidence Interval – Lower Bound	Confidence Interval – Upper Bound
**Indirect Effect – Early Period**				
(Intercept)	-8.221	0.707	-9.605	-6.836
hh_n_fully_vacc_nominal1	-0.301	0.076	-0.450	-0.151
hh_n_fully_vacc_nominal2	-1.263	0.054	-1.370	-1.157
hh_size	0.133	0.040	0.054	0.212
week_start2021-01-24	0.083	0.065	-0.044	0.210
week_start2021-01-31	0.273	0.076	0.124	0.423
week_start2021-02-07	0.376	0.083	0.214	0.539
week_start2021-02-14	0.445	0.100	0.249	0.640
week_start2021-02-21	0.769	0.100	0.573	0.965
week_start2021-02-28	1.018	0.100	0.822	1.215
week_start2021-03-07	0.543	0.115	0.318	0.767
week_start2021-03-14	0.001	0.125	-0.244	0.245
week_start2021-03-21	-0.525	0.141	-0.801	-0.249
male	-0.049	0.024	-0.095	-0.003
age_group1315	0.368	0.050	0.269	0.467
age_group1618	-30.787	17.001	-64.108	2.535
age_group36	0.083	0.038	0.009	0.157
age_group712	0.215	0.035	0.145	0.284
district_code2	0.097	0.136	-0.170	0.364
district_code4	0.128	0.092	-0.052	0.309
district_code5	0.321	0.090	0.144	0.498
district_code7	0.188	0.090	0.012	0.365
district_code9	0.234	0.086	0.066	0.403
district_code12	0.334	0.105	0.128	0.540
district_code15	0.171	0.092	-0.010	0.352
district_code16	0.905	0.268	0.381	1.430
city_type_rural	-0.013	0.097	-0.204	0.178
city_type_large_city	-0.044	0.062	-0.166	0.078
city_type_small_city	-0.076	0.055	-0.183	0.031
city_type_kibbutz	-0.287	0.163	-0.607	0.033
sector2	1.192	0.071	1.052	1.332
sector9	0.894	0.055	0.786	1.002
ses	-0.268	0.042	-0.351	-0.185
obese	0.158	0.044	0.072	0.245
active_malignancy	0.175	0.449	-0.705	1.056
diabetes	-0.100	0.173	-0.439	0.239
cvd_history	-0.120	0.226	-0.564	0.323
htn_history	0.446	0.243	-0.029	0.922
cpd_history	0.180	0.034	0.113	0.248
hh_n_males	-0.031	0.023	-0.076	0.013
hh_n_adult_smokers	-0.109	0.026	-0.159	-0.059
hh_n_obese	0.086	0.024	0.040	0.132
hh_pregnant	0.069	0.060	-0.048	0.186
hh_n_cvd	0.030	0.077	-0.121	0.182
hh_n_cpd	0.067	0.024	0.021	0.114
hh_n_dm	0.069	0.070	-0.069	0.206

hh_n_HTN	0.109	0.058	-0.004	0.221
hh_n_malig	-0.106	0.203	-0.503	0.291
hh_n_age_02	-0.102	0.044	-0.188	-0.015
hh_n_age_36	-0.106	0.041	-0.186	-0.025
hh_n_age_712	-0.079	0.042	-0.161	0.003
hh_n_age_1940	0.300	0.328	-0.343	0.942
hh_n_age_4160	0.180	0.328	-0.463	0.824
prevalence_02	-3.507	16.878	-36.588	29.573
prevalence_36	15.607	14.792	-13.384	44.598
prevalence_712	38.003	15.207	8.198	67.808
prevalence_1315	21.077	11.435	-1.337	43.490
prevalence_2564	1.554	18.961	-35.610	38.718
prevalence_6580	28.627	10.850	7.361	49.892
**Indirect Effect – Late Period**				
(Intercept)	-7.549	2.261	-11.980	-3.117
hh_n_booster_fully_vacc_nominal1	-0.233	0.057	-0.344	-0.121
hh_n_booster_fully_vacc_nominal2	-0.871	0.057	-0.983	-0.758
hh_size	-0.055	0.720	-1.467	1.357
week_start2021-07-18	0.584	0.184	0.224	0.944
week_start2021-07-25	0.705	0.181	0.350	1.060
week_start2021-08-01	1.196	0.173	0.857	1.535
week_start2021-08-08	1.355	0.172	1.018	1.693
week_start2021-08-15	1.543	0.177	1.196	1.890
week_start2021-08-22	1.651	0.184	1.291	2.011
week_start2021-08-29	1.731	0.190	1.358	2.103
week_start2021-09-05	1.818	0.188	1.450	2.185
week_start2021-09-12	1.798	0.186	1.433	2.163
week_start2021-09-19	1.346	0.188	0.977	1.715
male	0.022	0.020	-0.017	0.060
age_group36	0.488	0.032	0.425	0.552
age_group711	0.746	0.031	0.685	0.807
district_code2	0.533	0.093	0.351	0.715
district_code4	0.296	0.086	0.127	0.464
district_code5	0.542	0.083	0.379	0.705
district_code7	0.399	0.091	0.221	0.576
district_code9	0.371	0.086	0.202	0.540
district_code12	0.423	0.084	0.259	0.587
district_code15	0.366	0.088	0.194	0.538
district_code16	0.559	0.191	0.184	0.934
city_type_rural	-0.176	0.075	-0.323	-0.028
city_type_large_city	-0.030	0.054	-0.137	0.077
city_type_small_city	-0.025	0.046	-0.114	0.065
city_type_kibbutz	-0.099	0.094	-0.283	0.084
sector2	0.440	0.073	0.296	0.584
sector9	0.159	0.043	0.075	0.243
ses	-0.236	0.032	-0.298	-0.174
obese	0.048	0.040	-0.031	0.126
active_malignancy	0.077	0.357	-0.624	0.777
diabetes	-0.089	0.157	-0.396	0.218
cvd_history	-0.146	0.200	-0.538	0.245
htn_history	0.162	0.250	-0.327	0.652
cpd_history	0.076	0.029	0.018	0.133
hh_n_males	0.030	0.019	-0.007	0.066
hh_n_adult_smokers	0.012	0.020	-0.026	0.051

hh_n_obese	0.021	0.020	-0.018	0.060
hh_pregnant	0.111	0.054	0.006	0.217
hh_n_cvd	0.029	0.064	-0.097	0.155
hh_n_cpd	-0.011	0.021	-0.052	0.029
hh_n_dm	0.034	0.062	-0.088	0.156
hh_n_HTN	0.084	0.046	-0.005	0.174
hh_n_malig	0.079	0.151	-0.216	0.375
hh_n_age_02	0.139	0.721	-1.274	1.551
hh_n_age_36	0.237	0.720	-1.174	1.648
hh_n_age_1940	-0.290	0.306	-0.891	0.310
hh_n_age_4160	-0.335	0.307	-0.936	0.267
prevalence_02	-29.928	9.335	-48.225	-11.631
prevalence_36	-2.463	5.609	-13.456	8.530
prevalence_712	35.444	4.606	26.416	44.472
prevalence_1315	13.613	6.406	1.057	26.168
prevalence_2564	19.620	16.045	-11.828	51.067
prevalence_6580	-12.309	13.832	-39.420	14.802
hh_n_age_711	0.338	0.720	-1.073	1.750
hh_min_months_from_dose2	-0.004	0.030	-0.064	0.055
hh_max_months_from_dose2	0.112	0.028	0.057	0.167
**Direct Effect – Early Period**				
(Intercept)	-6.712	0.548	-7.785	-5.639
fully_vacc_nominal1	-2.878	0.098	-3.069	-2.687
hh_size	0.116	0.036	0.045	0.187
week_start2021-01-24	-0.028	0.053	-0.132	0.076
week_start2021-01-31	0.241	0.062	0.119	0.363
week_start2021-02-07	0.211	0.072	0.070	0.353
week_start2021-02-14	0.374	0.089	0.201	0.548
week_start2021-02-21	0.633	0.089	0.458	0.808
week_start2021-02-28	0.619	0.097	0.429	0.810
week_start2021-03-07	0.292	0.115	0.067	0.518
week_start2021-03-14	-0.303	0.135	-0.567	-0.039
week_start2021-03-21	-0.714	0.151	-1.011	-0.418
male	-0.020	0.027	-0.072	0.032
age_group4160	-0.113	0.037	-0.186	-0.040
age_group6180	0.034	0.453	-0.854	0.923
district_code2	0.061	0.127	-0.188	0.310
district_code4	0.094	0.088	-0.079	0.266
district_code5	0.135	0.083	-0.028	0.298
district_code7	0.251	0.083	0.088	0.414
district_code9	0.092	0.082	-0.068	0.253
district_code12	0.196	0.100	0.000	0.393
district_code15	0.098	0.082	-0.064	0.259
district_code16	0.500	0.300	-0.088	1.088
city_type_rural	0.028	0.092	-0.153	0.209
city_type_large_city	-0.017	0.057	-0.129	0.095
city_type_small_city	-0.063	0.049	-0.158	0.033
city_type_kibbutz	-0.466	0.204	-0.866	-0.067
sector2	0.863	0.062	0.742	0.984
sector9	0.492	0.046	0.402	0.582
ses	-0.285	0.039	-0.361	-0.208
pregnant	0.179	0.052	0.078	0.281
adult_smoker	-0.277	0.028	-0.331	-0.222
obese	0.094	0.033	0.030	0.158

active_malignancy	-0.029	0.234	-0.487	0.429
diabetes	-0.032	0.088	-0.204	0.140
cvd_history	0.048	0.087	-0.121	0.218
htn_history	0.165	0.060	0.047	0.282
cpd_history	-0.053	0.045	-0.141	0.036
hh_n_males	-0.032	0.019	-0.070	0.005
hh_n_adult_smokers	-0.068	0.027	-0.121	-0.016
hh_n_obese	0.063	0.023	0.017	0.108
hh_pregnant	0.145	0.056	0.036	0.255
hh_n_cvd	0.023	0.080	-0.133	0.180
hh_n_cpd	0.110	0.022	0.068	0.153
hh_n_dm	0.078	0.070	-0.060	0.216
hh_n_HTN	0.108	0.059	-0.007	0.223
hh_n_malig	-0.081	0.257	-0.584	0.422
hh_n_age_02	-0.032	0.039	-0.109	0.045
hh_n_age_36	-0.102	0.037	-0.175	-0.028
hh_n_age_712	-0.077	0.039	-0.153	-0.001
hh_n_age_1940	0.359	0.500	-0.622	1.339
hh_n_age_4160	0.269	0.501	-0.713	1.250
prevalence_02	-17.408	16.290	-49.335	14.518
prevalence_36	-8.017	17.852	-43.007	26.973
prevalence_712	39.585	12.310	15.457	63.713
prevalence_1315	0.232	10.594	-20.531	20.995
prevalence_2564	42.021	17.891	6.956	77.087
prevalence_6580	7.998	11.234	-14.020	30.015
hh_n_fully_vacc	-0.321	0.067	-0.453	-0.189
**Direct Effect – Late Period**				
(Intercept)	-8.488	0.488	-9.444	-7.532
booster_fully_vacc_nominal1	-1.987	0.096	-2.174	-1.799
hh_size	0.304	0.022	0.261	0.348
week_start2021-07-18	0.451	0.213	0.035	0.868
week_start2021-07-25	0.891	0.200	0.499	1.283
week_start2021-08-01	1.140	0.194	0.759	1.521
week_start2021-08-08	1.438	0.192	1.061	1.815
week_start2021-08-15	1.792	0.196	1.407	2.177
week_start2021-08-22	1.981	0.204	1.581	2.381
week_start2021-08-29	2.096	0.212	1.681	2.510
week_start2021-09-05	1.992	0.210	1.579	2.404
week_start2021-09-12	1.953	0.209	1.543	2.362
week_start2021-09-19	1.693	0.211	1.279	2.108
male	-0.128	0.028	-0.183	-0.072
age_group4160	-0.051	0.037	-0.124	0.022
age_group6180	1.085	0.369	0.361	1.809
district_code2	0.480	0.102	0.281	0.679
district_code4	0.331	0.095	0.145	0.517
district_code5	0.478	0.092	0.297	0.658
district_code7	0.424	0.103	0.223	0.626
district_code9	0.367	0.096	0.178	0.555
district_code12	0.407	0.095	0.222	0.593
district_code15	0.300	0.099	0.106	0.493
district_code16	0.797	0.201	0.403	1.191
city_type_rural	-0.122	0.084	-0.286	0.041
city_type_large_city	0.061	0.060	-0.056	0.179

city_type_small_city	0.018	0.052	-0.085	0.121
city_type_kibbutz	-0.211	0.113	-0.432	0.010
sector2	0.698	0.081	0.540	0.856
sector9	0.499	0.051	0.400	0.599
ses	-0.242	0.035	-0.310	-0.174
pregnant	0.230	0.060	0.112	0.348
adult_smoker	-0.125	0.027	-0.178	-0.072
obese	0.004	0.036	-0.067	0.075
active_malignancy	0.317	0.185	-0.046	0.681
diabetes	-0.120	0.102	-0.321	0.080
cvd_history	-0.003	0.094	-0.187	0.182
htn_history	0.157	0.061	0.037	0.278
cpd_history	0.021	0.043	-0.064	0.106
hh_n_males	0.000	0.019	-0.038	0.038
hh_n_adult_smokers	-0.022	0.026	-0.074	0.029
hh_n_obese	-0.039	0.026	-0.089	0.011
hh_pregnant	0.174	0.068	0.040	0.307
hh_n_cvd	-0.068	0.086	-0.237	0.101
hh_n_cpd	0.053	0.022	0.010	0.097
hh_n_dm	-0.008	0.081	-0.167	0.151
hh_n_HTN	0.060	0.061	-0.059	0.180
hh_n_malig	0.167	0.166	-0.159	0.492
hh_n_age_02	-0.130	0.028	-0.185	-0.075
hh_n_age_36	-0.059	0.025	-0.108	-0.010
hh_n_age_1940	-0.524	0.417	-1.342	0.294
hh_n_age_4160	-0.559	0.418	-1.378	0.259
prevalence_02	-15.539	10.754	-36.617	5.540
prevalence_36	5.441	6.311	-6.927	17.810
prevalence_712	20.321	5.919	8.720	31.921
prevalence_1315	5.337	8.449	-11.224	21.898
prevalence_2564	-0.088	18.443	-36.236	36.059
prevalence_6580	-3.544	15.714	-34.344	27.255
hh_min_months_from_dose2	0.017	0.120	-0.217	0.252
hh_max_months_from_dose2	0.099	0.122	-0.140	0.338
hh_n_booster_fully_vacc	-0.537	0.072	-0.679	-0.396
**Secondary Attack Rate – Early Period**				
(Intercept)	-28.623	754.033	NA	49.476
hh_size	0.243	0.125	-0.006	0.486
hh_first_covid_city_type_rural	-0.458	0.322	-1.120	0.152
hh_first_covid_city_type_large_city	-0.002	0.179	-0.351	0.352
hh_first_covid_city_type_small_city	-0.093	0.161	-0.406	0.224
hh_first_covid_city_type_kibbutz	-0.092	0.694	-1.641	1.167
hh_first_covid_ses	-0.222	0.123	-0.466	0.015
hh_first_covid_district_code2	-0.233	0.332	-0.907	0.401
hh_first_covid_district_code4	0.075	0.207	-0.332	0.480
hh_first_covid_district_code5	-0.069	0.215	-0.493	0.353
hh_first_covid_district_code7	0.109	0.199	-0.281	0.500
hh_first_covid_district_code9	0.050	0.204	-0.350	0.449
hh_first_covid_district_code12	0.161	0.210	-0.252	0.573
hh_first_covid_district_code15	0.159	0.228	-0.290	0.605
hh_first_covid_district_code16	2.498	0.925	0.795	4.581
hh_first_covid_sector2	0.804	0.211	0.389	1.215
hh_first_covid_sector9	0.807	0.148	0.518	1.099
hh_total_n_males	-0.045	0.065	-0.172	0.081

hh_total_n_adult_smokers	-0.036	0.078	-0.190	0.117
hh_total_n_obese	0.045	0.071	-0.095	0.182
hh_total_n_cvd	-0.270	0.232	-0.747	0.166
hh_total_n_cpd	0.092	0.066	-0.040	0.221
hh_total_n_dm	-0.209	0.214	-0.647	0.195
hh_total_n_HTN	0.375	0.173	0.029	0.711
hh_total_n_malig	0.539	0.544	-0.620	1.586
hh_total_n_02	-0.109	0.137	-0.375	0.162
hh_total_n_36	-0.137	0.129	-0.388	0.118
hh_total_n_712	-0.012	0.134	-0.271	0.253
hh_total_n_1940	13.179	377.017	-25.892	NA
hh_total_n_4160	13.027	377.017	-26.026	NA
week_start2021-01-24	0.163	0.175	-0.181	0.506
week_start2021-01-31	0.134	0.176	-0.212	0.480
week_start2021-02-07	-0.006	0.193	-0.386	0.369
week_start2021-02-14	0.363	0.199	-0.030	0.752
week_start2021-02-21	0.115	0.213	-0.308	0.530
week_start2021-02-28	-0.024	0.236	-0.496	0.433
week_start2021-03-07	-0.416	0.287	-1.002	0.129
week_start2021-03-14	-0.464	0.432	-1.392	0.325
pregnant	-0.139	0.214	-0.571	0.269
hh_pregnant	-0.403	0.250	-0.914	0.069
hh_n_fully_vacc	0.174	0.261	-0.351	0.673
index_fully_vacc1	-1.276	0.448	-2.233	-0.456
**Secondary Attack Rate – Late Period**				
(Intercept)	-28.611	716.711	NA	53.023
hh_first_covid_months_from_dose2	0.029	0.088	-0.143	0.202
hh_size	0.361	0.083	0.199	0.524
hh_first_covid_city_type_rural	-0.320	0.279	-0.874	0.221
hh_first_covid_city_type_large_city	-0.216	0.197	-0.601	0.172
hh_first_covid_city_type_small_city	0.036	0.178	-0.312	0.388
hh_first_covid_city_type_kibbutz	-0.355	0.411	-1.193	0.427
hh_first_covid_ses	0.001	0.111	-0.217	0.219
hh_first_covid_district_code2	0.349	0.293	-0.223	0.926
hh_first_covid_district_code4	-0.022	0.269	-0.547	0.510
hh_first_covid_district_code5	-0.172	0.259	-0.676	0.342
hh_first_covid_district_code7	-0.237	0.253	-0.730	0.266
hh_first_covid_district_code9	-0.106	0.257	-0.607	0.403
hh_first_covid_district_code12	0.287	0.255	-0.208	0.793
hh_first_covid_district_code15	0.099	0.285	-0.456	0.661
hh_first_covid_district_code16	0.792	0.550	-0.294	1.885
hh_first_covid_sector2	-0.033	0.318	-0.668	0.582
hh_first_covid_sector9	0.532	0.189	0.165	0.908
hh_total_n_males	0.018	0.070	-0.119	0.156
hh_total_n_adult_smokers	-0.081	0.077	-0.233	0.070
hh_total_n_obese	0.045	0.082	-0.116	0.204
hh_total_n_cvd	-0.167	0.253	-0.683	0.315
hh_total_n_cpd	-0.040	0.076	-0.189	0.107
hh_total_n_dm	-0.217	0.280	-0.787	0.316
hh_total_n_HTN	0.059	0.183	-0.306	0.412
hh_total_n_malig	0.474	0.457	-0.454	1.359
hh_total_n_02	-0.221	0.103	-0.423	-0.020
hh_total_n_36	0.111	0.094	-0.072	0.295
hh_total_n_1940	12.628	358.355	-28.402	NA

hh_total_n_4160	12.621	358.355	-28.390	NA
week_start2021-07-18	-0.451	0.725	-1.881	1.004
week_start2021-07-25	-0.353	0.626	-1.546	0.944
week_start2021-08-01	0.118	0.587	-0.986	1.355
week_start2021-08-08	0.006	0.577	-1.076	1.226
week_start2021-08-15	0.330	0.562	-0.719	1.526
week_start2021-08-22	0.315	0.566	-0.741	1.517
week_start2021-08-29	0.254	0.567	-0.804	1.458
week_start2021-09-05	0.446	0.570	-0.621	1.656
week_start2021-09-12	0.110	0.582	-0.981	1.339
week_start2021-09-19	-0.069	0.609	-1.220	1.204
pregnant	-0.131	0.250	-0.638	0.346
hh_pregnant	-0.275	0.277	-0.839	0.251
hh_n_booster_fully_vacc	-0.417	0.368	-1.181	0.274
hh_min_months_from_dose2	0.061	0.096	-0.127	0.248
hh_max_months_from_dose2	NA	NA	NA	NA
index_fully_vacc1	-1.588	0.424	-2.504	-0.820

Caption: Output summaries from the models used in the study.

References and Notes

1. N. Zhu, D. Zhang, W. Wang, X. Li, B. Yang, J. Song, X. Zhao, B. Huang, W. Shi, R. Lu, P. Niu, F. Zhan, X. Ma, D. Wang, W. Xu, G. Wu, G. F. Gao, W. Tan, China Novel Coronavirus Investigating and Research Team, A Novel Coronavirus from Patients with Pneumonia in China, 2019. *N. Engl. J. Med.* **382**, 727–733 (2020). doi:10.1056/NEJMoa2001017 Medline

2. Center for Systems Science and Engineering (CSSE) at Johns Hopkins University (JHU), "COVID-19 Dashboard" (2022); https://coronavirus.jhu.edu/map.html.

3. M. E. Halloran, M. Haber, I. M. Longini Jr., C. J. Struchiner, Direct and indirect effects in vaccine efficacy and effectiveness. *Am. J. Epidemiol.* **133**, 323–331 (1991). doi:10.1093/oxfordjournals.aje.a115884 Medline

4. Z. Wang, W. Ma, X. Zheng, G. Wu, R. Zhang, Household transmission of SARS-CoV-2. *J. Infect.* **81**, 179–182 (2020). doi:10.1016/j.jinf.2020.03.040 Medline

5. K. Danis, O. Epaulard, T. Bénet, A. Gaymard, S. Campoy, E. Botelho-Nevers, M. Bouscambert-Duchamp, G. Spaccaferri, F. Ader, A. Mailles, Z. Boudalaa, V. Tolsma, J. Berra, S. Vaux, E. Forestier, C. Landelle, E. Fougere, A. Thabuis, P. Berthelot, R. Veil, D. Levy-Bruhl, C. Chidiac, B. Lina, B. Coignard, C. Saura, Investigation Team, Cluster of Coronavirus Disease 2019 (COVID-19) in the French Alps, February 2020. *Clin. Infect. Dis.* **71**, 825–832 (2020). doi:10.1093/cid/ciaa424 Medline

6. I. Dattner, Y. Goldberg, G. Katriel, R. Yaari, N. Gal, Y. Miron, A. Ziv, R. Sheffer, Y. Hamo, A. Huppert, The role of children in the spread of COVID-19: Using household data from Bnei Brak, Israel, to estimate the relative susceptibility and infectivity of children. *PLOS Comput. Biol.* **17**, e1008559 (2021). doi:10.1371/journal.pcbi.1008559 Medline

7. Z. J. Madewell, Y. Yang, I. M. Longini Jr., M. E. Halloran, N. E. Dean, Factors Associated With Household Transmission of SARS-CoV-2: An Updated Systematic Review and Meta-analysis. *JAMA Netw. Open* **4**, e2122240 (2021). doi:10.1001/jamanetworkopen.2021.22240 Medline

8. F. P. Polack, S. J. Thomas, N. Kitchin, J. Absalon, A. Gurtman, S. Lockhart, J. L. Perez, G. Pérez Marc, E. D. Moreira, C. Zerbini, R. Bailey, K. A. Swanson, S. Roychoudhury, K. Koury, P. Li, W. V. Kalina, D. Cooper, R. W. Frenck Jr., L. L. Hammitt, Ö. Türeci, H. Nell, A. Schaefer, S. Ünal, D. B. Tresnan, S. Mather, P. R. Dormitzer, U. Şahin, K. U. Jansen, W. C. Gruber, C4591001 Clinical Trial Group, Safety and Efficacy of the BNT162b2 mRNA Covid-19 Vaccine. *N. Engl. J. Med.* **383**, 2603–2615 (2020). doi:10.1056/NEJMoa2034577 Medline

9. N. Dagan, N. Barda, E. Kepten, O. Miron, S. Perchik, M. A. Katz, M. A. Hernán, M. Lipsitch, B. Reis, R. D. Balicer, BNT162b2 mRNA Covid-19 Vaccine in a Nationwide Mass Vaccination Setting. *N. Engl. J. Med.* **384**, 1412–1423 (2021). doi:10.1056/NEJMoa2101765 Medline

10. E. J. Haas, F. J. Angulo, J. M. McLaughlin, E. Anis, S. R. Singer, F. Khan, N. Brooks, M. Smaja, G. Mircus, K. Pan, J. Southern, D. L. Swerdlow, L. Jodar, Y. Levy, S. Alroy-Preis, Impact and effectiveness of mRNA BNT162b2 vaccine against SARS-CoV-2 infections and COVID-19 cases, hospitalisations, and deaths following a nationwide

vaccination campaign in Israel: An observational study using national surveillance data. *Lancet* **397**, 1819–1829 (2021). doi:10.1016/S0140-6736(21)00947-8 Medline

11. J. Salo, M. Hägg, M. Kortelainen, T. Leino, T. Saxell, M. Siikanen, L. Sääksvuori, The indirect effect of mRNA-based Covid-19 vaccination on unvaccinated household members. medRxiv 2021.05.27.21257896 [Preprint] (2021). https://doi.org/10.1101/2021.05.27.21257896.

12. M. Layan, M. Gilboa, T. Gonen, M. Goldenfeld, L. Meltzer, A. Andronico, N. Hozé, S. Cauchemez, G. Regev-Yochay, Impact of BNT162b2 vaccination and isolation on SARS-CoV-2 transmission in Israeli households: An observational study. medRxiv 2021.07.12.21260377 [Preprint] (2021). https://doi.org/10.1101/2021.07.12.21260377.

13. O. Milman, I. Yelin, N. Aharony, R. Katz, E. Herzel, A. Ben-Tov, J. Kuint, S. Gazit, G. Chodick, T. Patalon, R. Kishony, Community-level evidence for SARS-CoV-2 vaccine protection of unvaccinated individuals. *Nat. Med.* **27**, 1367–1369 (2021). doi:10.1038/s41591-021-01407-5 Medline

14. Israel Ministry of Health, "Israel COVID-19 Data Tracker" (2022); https://datadashboard.health.gov.il/COVID-19/general.

15. European Centre for Disease Prevention and Control, "COVID-19 Vaccine Tracker" (2022); https://vaccinetracker.ecdc.europa.eu/public/extensions/Covid-19/vaccine-tracker.html.

16. Mayo Clinic, "U.S. COVID-19 Vaccine Tracker" (2022); www.mayoclinic.org/coronavirus-covid-19/vaccine-tracker.

17. T. J. Vanderweele, E. J. Tchetgen Tchetgen, M. E. Halloran, Components of the indirect effect in vaccine trials: Identification of contagion and infectiousness effects. *Epidemiology* **23**, 751–761 (2012). doi:10.1097/EDE.0b013e31825fb7a0 Medline

18. A. Richterman, E. A. Meyerowitz, M. Cevik, Indirect Protection by Reducing Transmission: Ending the Pandemic with Severe Acute Respiratory Syndrome Coronavirus 2 Vaccination. *Open Forum Infect. Dis.* **9**, ofab259 (2022). doi:10.1093/ofid/ofab259

19. N. Barda, N. Dagan, C. Cohen, M. A. Hernán, M. Lipsitch, I. S. Kohane, B. Y. Reis, R. D. Balicer, Effectiveness of a third dose of the BNT162b2 mRNA COVID-19 vaccine for preventing severe outcomes in Israel: An observational study. *Lancet* **398**, 2093–2100 (2021). doi:10.1016/S0140-6736(21)02249-2 Medline

20. M. Lipsitch, E. Tchetgen Tchetgen, T. Cohen, Negative controls: A tool for detecting confounding and bias in observational studies. *Epidemiology* **21**, 383–388 (2010). doi:10.1097/EDE.0b013e3181d61eeb Medline

21. X. Shi, W. Miao, E. T. Tchetgen, A Selective Review of Negative Control Methods in Epidemiology. *Curr. Epidemiol. Rep.* **7**, 190–202 (2020). doi:10.1007/s40471-020-00243-4 Medline

22. L. S. Shekerdemian, N. R. Mahmood, K. K. Wolfe, B. J. Riggs, C. E. Ross, C. A. McKiernan, S. M. Heidemann, L. C. Kleinman, A. I. Sen, M. W. Hall, M. A. Priestley, J. K. McGuire, K. Boukas, M. P. Sharron, J. P. Burns, International COVID-19 PICU Collaborative, Characteristics and Outcomes of Children With Coronavirus Disease 2019

(COVID-19) Infection Admitted to US and Canadian Pediatric Intensive Care Units. *JAMA Pediatr.* **174**, 868–873 (2020). doi:10.1001/jamapediatrics.2020.1948 Medline

23. J. Toubiana, C. Poirault, A. Corsia, F. Bajolle, J. Fourgeaud, F. Angoulvant, A. Debray, R. Basmaci, E. Salvador, S. Biscardi, P. Frange, M. Chalumeau, J.-L. Casanova, J. F. Cohen, S. Allali, Kawasaki-like multisystem inflammatory syndrome in children during the covid-19 pandemic in Paris, France: Prospective observational study. *BMJ* **369**, m2094 (2020). doi:10.1136/bmj.m2094 Medline

24. J. F. Ludvigsson, Case report and systematic review suggest that children may experience similar long-term effects to adults after clinical COVID-19. *Acta Paediatr.* **110**, 914–921 (2021). doi:10.1111/apa.15673 Medline

25. A. Mastrangelo, W. Morello, E. Vidal, I. Guzzo, L. Annicchiarico Petruzzelli, E. Benetti, M. Materassi, M. Giordano, A. Pasini, C. Corrado, G. Puccio, R. Chimenz, C. Pecoraro, L. Massella, L. Peruzzi, G. Montini, COVID-19 Task Force of the Italian Society of Pediatric Nephrology, Impact of COVID-19 Pandemic in Children with CKD or Immunosuppression. *Clin. J. Am. Soc. Nephrol.* **16**, 449–451 (2021). doi:10.2215/CJN.13120820 Medline

26. E. Goldstein, K. Paur, C. Fraser, E. Kenah, J. Wallinga, M. Lipsitch, Reproductive numbers, epidemic spread and control in a community of households. *Math. Biosci.* **221**, 11–25 (2009). doi:10.1016/j.mbs.2009.06.002 Medline

27. E. K. Accorsi, X. Qiu, E. Rumpler, L. Kennedy-Shaffer, R. Kahn, K. Joshi, E. Goldstein, M. J. Stensrud, R. Niehus, M. Cevik, M. Lipsitch, How to detect and reduce potential sources of biases in studies of SARS-CoV-2 and COVID-19. *Eur. J. Epidemiol.* **36**, 179–196 (2021). doi:10.1007/s10654-021-00727-7 Medline

28. S. Hayek, G. Shaham, Y. Ben-Shlomo, E. Kepten, N. Dagan, D. Nevo, M. Lipsitch, B. Reis, R. Balicer, N. Barda, Indirect protection of children from SARS-CoV-2 infection through parental vaccination, Zenodo (2022); https://doi.org/10.5281/zenodo.5883892.

29. M. A. Hernán, J. M. Robins, Using Big Data to Emulate a Target Trial When a Randomized Trial Is Not Available. *Am. J. Epidemiol.* **183**, 758–764 (2016). doi:10.1093/aje/kwv254 Medline

30. M. E. Halloran, C. J. Struchiner, Study designs for dependent happenings. *Epidemiology* **2**, 331–338 (1991). doi:10.1097/00001648-199109000-00004 Medline

31. M. E. Halloran, The Minicommunity Design to Assess Indirect Effects of Vaccination. *Epidemiol. Methods* **1**, 83–105 (2012). doi:10.1515/2161-962X.1008 Medline

32. L. N. Yelland, A. B. Salter, P. Ryan, Performance of the modified Poisson regression approach for estimating relative risks from clustered prospective data. *Am. J. Epidemiol.* **174**, 984–992 (2011). doi:10.1093/aje/kwr183 Medline

33. G. Y. Zou, A. Donner, Extension of the modified Poisson regression model to prospective studies with correlated binary data. *Stat. Methods Med. Res.* **22**, 661–670 (2013). doi:10.1177/0962280211427759 Medline

34. G. Zou, A modified poisson regression approach to prospective studies with binary data. *Am. J. Epidemiol.* **159**, 702–706 (2004). doi:10.1093/aje/kwh090 Medline

35. D. L. Miglioretti, P. J. Heagerty, Marginal modeling of nonnested multilevel data using standard software. *Am. J. Epidemiol.* **165**, 453–463 (2007). doi:10.1093/aje/kwk020 Medline

36. S. Seaman, M. Pavlou, A. Copas, Review of methods for handling confounding by cluster and informative cluster size in clustered data. *Stat. Med.* **33**, 5371–5387 (2014). doi:10.1002/sim.6277 Medline
